# The Transcriptional Response to DNA-Double-Strand Breaks in *Physcomitrella patens*

**DOI:** 10.1371/journal.pone.0161204

**Published:** 2016-08-18

**Authors:** Yasuko Kamisugi, John W. Whitaker, Andrew C. Cuming

**Affiliations:** 1 Centre for Plant Sciences, Faculty of Biological Sciences, Leeds University, Leeds LS2 9JT, United Kingdom; 2 Bioinformatics Research Group, School of Molecular and Cellular Biology, Leeds University, Leeds LS2 9JT, United Kingdom; Institut Pasteur, FRANCE

## Abstract

The model bryophyte *Physcomitrella patens* is unique among plants in supporting the generation of mutant alleles by facile homologous recombination-mediated gene targeting (GT). Reasoning that targeted transgene integration occurs through the capture of transforming DNA by the homology-dependent pathway for DNA double-strand break (DNA-DSB) repair, we analysed the genome-wide transcriptomic response to bleomycin-induced DNA damage and generated mutants in candidate DNA repair genes. Massively parallel (Illumina) cDNA sequencing identified potential participants in gene targeting. Transcripts encoding DNA repair proteins active in multiple repair pathways were significantly up-regulated. These included Rad51, CtIP, DNA ligase 1, Replication protein A and ATR in homology-dependent repair, Xrcc4, DNA ligase 4, Ku70 and Ku80 in non-homologous end-joining and Rad1, Tebichi/polymerase theta, PARP in microhomology-mediated end-joining. Differentially regulated cell-cycle components included up-regulated Rad9 and Hus1 DNA-damage-related checkpoint proteins and down-regulated D-type cyclins and B-type CDKs, commensurate with the imposition of a checkpoint at G_2_ of the cell cycle characteristic of homology-dependent DNA-DSB repair. Candidate genes, including ATP-dependent chromatin remodelling helicases associated with repair and recombination, were knocked out and analysed for growth defects, hypersensitivity to DNA damage and reduced GT efficiency. Targeted knockout of *PpCtIP*, a cell-cycle activated mediator of homology-dependent DSB resection, resulted in bleomycin-hypersensitivity and greatly reduced GT efficiency.

## Introduction

The moss *Physcomitrella patens* is the pre-eminent experimental model for comparative analysis of the evolution of gene function in plants. As a bryophyte, *P*. *patens* occupies a basal position in the land plant phylogeny. The bryophytes diverged from the land plant lineage approximately 450–500 million years ago and were the first group of plants to colonise terrestrial habitats [1, 2]. Many of the features present in extant bryophytes represent ancient adaptations necessary for the conquest of dry land, including properties of resilience to a wide range of abiotic stresses. Experimentally, *P*. *patens* is highly amenable to genetic analysis and manipulation. The determination of the complete genome sequence of the moss and the development of a well-marked sequence-anchored linkage map provide the opportunity for the forward-genetic identification of genes responsible for key developmental transitions and responses to environmental and hormonal cues [3, 4]. Most significantly, *P*. *patens* has emerged as an excellent model for the reverse-genetic analysis of gene function due to its remarkable ability to integrate transgenes at predefined loci through homologous recombination-mediated “gene targeting” (GT) [5, 6, 7].

Gene targeting in *P*. *patens* enables precise allele replacement at high frequency. Only relatively short (500-1000bp) lengths of homology are required for efficient GT, so that a range of gene modifications are possible [6]. These include gene disruption and deletion (gene knockout), precise insertion of reporter genes or epitope and affinity tags to native loci (gene knock-in), and sequence alteration by as little as a single base (directed point mutation). Such efficient GT is not possible in other model plant species. Alternative approaches such as stringent counter-selection to recover low frequency targeting events [8] or the deployment of complex protein engineering procedures to design site specific endonucleases capable of introducing DNA breaks at selected sites for transgene insertion have been described [9], but currently remain of limited use for plant genetic manipulation. Targeted mutagenesis through inaccurate repair of CRISPR/Cas9-induced DNA breaks can be used to generate mutant alleles [10], but its potential to enable high-frequency precision gene editing is uncertain and likely to be limited by the low frequency with which homology-dependent repair occurs in angiosperms.

The insertion of transgenes in the genomes of eukaryotic organisms is believed to occur through the capture of transforming DNA by the endogenous mechanisms of DNA double-strand break (DNA-DSB) repair [11]. DNA-DSBs occur with high frequency as a result of exposure to environmental insults such as ionising radiation or chemical mutagens, and (most commonly) through the frequent collapse of replication forks during DNA synthesis, when the replication machinery encounters a single-strand break. It is therefore essential that organisms deploy a range of efficient procedures to repair DNA-DSBs if they are not to suffer catastrophic consequences of genetic loss. There are three principal paths by which DNA-DSBs are repaired. These are the non-homologous end-joining (NHEJ) pathway, the microhomology-mediated end-joining pathway (MMEJ) and the homology-dependent pathway (homologous recombination: HR). NHEJ is typically activated during the G_1_ phase of the cell-cycle, when the ATM protein kinase initiates a phosphorylation-based signalling cascade culminating in a cell-cycle checkpoint [12]. The broken ends are successively bound by the proteins Ku70/Ku80, and religated through the action of DNA ligase 4. This mechanism appears to be highly conserved throughout the Eukaryota. HR is activated during S and G_2_, and involves resection of one strand of the broken DNA to leave a long 3’-single-stranded overhang. The ATR protein kinase induces a G_2_-specific cell-cycle checkpoint [12], and the single-strand end is successively modified by protein interactions, finally becoming coated with the Rad51 recombinase–the eukaryotic homologue of the *E*. *coli* RecA protein–to form an invasive nucleoprotein strand that can invade a complementary sequence (usually the adjacent, undamaged, replicated strand) that acts as a template for the accurate resynthesis of the damaged DNA [13]. MMEJ also occurs principally during S-phase. This is a rapid but highly inaccurate mechanism, the broken ends being processed by only a short resection, and the end-joining characterised by insertions or deletions [14].

When a transgene is delivered to a cell, multiple copies of exogenous DNA are taken up, and free ends of these molecules are most likely recognised by endogenous DNA repair enzymes that incorporate the transgenic sequence into the genome. The fate of the transforming DNA will depend on the dominant DNA repair pathway operating in the target cell. Thus in yeasts (*S*. *cerevisiae* and *S*. *pombe*) the HR-DNA repair pathways are highly active, and transgenes will be integrated at targeted sites, if they contain short lengths of homologous sequence [15]. By contrast, transgene integration into the genomes of flowering plants occurs essentially at random, even when transgenes contain long (several kilobases) of genome homology. This most likely reflects a dominant NHEJ pathway capturing the incoming DNA and inserting it adventitiously at available DSBs [11].

Understanding how HR-mediated transgene insertion occurs in plant cells would be of value in enabling the development of knowledge-based strategies to increase the frequency of GT for genetic manipulation of flowering plants–particularly in the development of precision-engineered crop species. To this end, it is important to study the process of HR-mediated DNA repair and transgene capture in a model plant species that is highly competent to undertake this process. *P*. *patens* is such a model [7, 16]. In order to identify potential components of the plant HR-mediated DNA repair/GT pathway, we have undertaken a genome-wide analysis of the transcriptional response to the induction of DNA-DSBs in *P*. *patens*. This identifies a number of candidate genes whose contribution to efficient GT can now be tested.

## Materials and Methods

### Plant material

*Physcomitrella patens* ssp. *patens* (accession Villersexel K3) [4,17] was vegetatively propagated as protonemal homogenates on cellophane-overlaid BCD agar medium supplemented with ammonium tartrate (BCDAT) or as individual plants regenerated from protonemal tissue explants (“spot inocula”) on BCDAT-agar as described previously[18].

Responses to DNA damage were measured following either chronic or acute treatment with Bleomycin (Bleocin inj., Euro Nippon Kayaku GmbH, Germany). Chronic treatment was by growth of explants on BCDAT-agar containing bleomycin at concentrations indicated in the text. For acute treatment, protonemal tissue (7d following subculture) was harvested from cellophane overlays, briefly blotted with sterile filter paper to remove residual water, then dispersed in 5ml liquid BCDAT medium containing bleomycin as indicated. Recovery from acute treatment was measured by inoculating explants on drug-free BCDAT agar.

Sensitivity to bleomycin treatment was assessed by measuring the rate of plant growth as previously described [19]. Digital photographs of plants were taken at intervals following inoculation, and the area of each plant determined as the number of pixels using the ImageJ suite of image analysis programs [20]. Variation between photographs was minimised by normalising the area occupied by each plant with relative to the area of the culture dish in each image to generate a “growth index”.

### Gene targeting

For gene targeted knockouts of helicases, sequences comprising approx. 1kb from each of the 5’- and 3’- sequences of target genes were amplified by PCR and cloned into *EcoR*V and *Ecl*136II sites, located on either side of an *NPTII* selection cassette by blunt-end ligation in a pMBL5-derived [6] plasmid vector. For the *PpCtIP* gene, a 3.9 kb fragment comprising the entire gene was amplified and the selection cassette was cloned between *Mun*I and *Cla*I sites, thereby replacing 1018bp of the 1335bp polypeptide coding sequence. Linear targeting fragments were amplified by PCR for delivery to protoplasts. Primers used are listed in S1 Table. DNA delivery, regeneration of protoplasts, selection of transformants on medium containing G418 and analysis of targeted transformants by PCR and Southern blot analysis were all carried out as previously described [6]. The nature and verification of mutant genotypes is provided in S1–S7 Figs. The ability of wild-type and mutant strains to undertake targeted transgene integration was determined by targeted knockout of the *PpAPT* locus (Pp3c8_16590V3.1), encoding adenosine phosphoribosyl transferase. Targeted replacement of this gene confers resistance to the nucleotide analogue 2-fluoroadenine (2-FA). Protoplasts were transformed with a *PpAPT* targeting construct comprising 1419bp (5’) and 1001bp (3’) genomic sequences flanking a 35S-driven hygromycin resistance cassette, cloned between two *Xba*I sites located in intron 4 and exon 5 of the *PpAPT* gene, respectively. The proportion of hygromycin-resistant transformants exhibiting 2-FA resistance represented the gene targeting frequency [21].

### RNA extraction and analysis

Total RNA was extracted from protonemal tissue propagated for 7d following subculture using aqueous phenol extraction and selective precipitation with 2.5M NaCl as previously described [18].

For the isolation of RNA from a polyribosome-enriched fraction, protonemal tissue was harvested and residual liquid was squeezed from the tissue by pressing between two layers of Whatman 3MM chromatography paper. Squeeze-dried tissue was frozen in liquid nitrogen and homogenized. Three replicates of approx. 0.4g squeeze-dried protonemata, each corresponding to four 9cm Petri dish cultures were ground to a powder in liquid N_2_ in a mortar and pestle and homogenised in 30 ml 200mM sucrose– 200mM Tris-Cl, pH8.5 – 60mM KCl– 30mM MgCl_2_−1% (v/v) Triton X-100 – 5mM β-mercaptoethanol. The homogenates were centrifuged (20 min x 30,000 *x g*: Sorvall RC5B SS-34 rotor) and the supernatants aspirated and layered over 5ml sucrose cushions (1M sucrose– 40mM Tris-Cl, pH8.5 – 20mM KCl– 10mM MgCl_2_) and centrifuged at 150,000 x *g* (3h, Beckman Sw28 rotor). Following centrifugation, the pellets were each resuspended in 0.5 ml RNA extraction buffer for isolation of RNA by phenol-chloroform extraction.

All RNA samples were dissolved in sterile water and digested with 1 unit RQ Dnase 1 (Promega) (10’ at room temperature) in a total volume of 60 microlitres before addition of Na_2_EDTA to 5mM and a further phenol-chloroform extraction. The final pellets were dissolved in sterile water for quantification using a Nanodrop spectrophotometer, and the integrity of the RNA monitored by electrophoretic analysis.

For gene expression profiling by quantitative real-time PCR, RNA (1μg) was reverse-transcribed using a Promega reverse transcription system. The 20μl reaction mixture was diluted 25-fold and 2.5 μl aliquots were used for real-time PCR amplifications (10μl) used the KAPA Biosystems SybrFast reaction kit in a BioRad CFX96 Real Time Detection System. Three biological replicate samples were analysed in duplicate. Transcript abundance was estimated by reference to both internal and external reference sequences. As an external reference, *Physcomitrella* RNA samples were “spiked” with tenfold serial dilutions (10^−1^–10^−4^) of an *in vitro* transcript from a full-length wheat “Em” cDNA [22, 23] prior to reverse transcription. These were used to test a number of candidate internal reference sequences, corresponding to *Physcomitrella* gene models Pp3c10_13820V3.1 (SAND family endocytosis protein), Pp3c27_2250V3.1 (Clathrin adapter complex subunit: “CAP-50”)), Pp3c4_32050V3.1 (Acyltransferase) and Pp3c13_7750V3.1 (Ribosomal protein S4) for stability of expression in response to bleomycin treatment. CAP-50 was found to have the most stable expression levels under all conditions tested and was subsequently used as the internal reference standard throughout. Primers used for PCR amplification are detailed in S2 Table.

Illumina mRNA-sequencing was used for digital expression analysis of the response to bleomycin treatment. Polyribosome-enriched RNA derived from three replicate control and bleomycin-treated tissue samples was used. For each treatment, equal quantities of the three replicate samples were mixed and submitted for Illumina short-read (36 base) sequence analysis by GATC Biotech AG, Konstanz, Germany. Aliquots (1μg) were used for cDNA synthesis using the Clontech SMART cDNA protocol (Clontech Protocol No. PT3041-1; Cat No. 634902). Libraries were prepared according to the Illumina protocol accompanying the DNA Sample Kit (Cat No. 0801–0303). Briefly, DNA was end-repaired using a combination of T4 DNA polymerase, *E*. *coli* DNA Pol I large fragment (Klenow polymerase) and T4 polynucleotide kinase. The blunt, phosphorylated ends were treated with Klenow polymerase and dATP to yield a protruding 3’- 'A' base for ligation of the Illumina adapters which have a single 'T' base overhang at the 3’ end. After adapter ligation, DNA was PCR-amplified with Illumina primers for 12 cycles and library fragments of ~375 bp (insert plus adaptor and PCR primer sequences) were band-isolated from an agarose gel. The purified DNA was captured on an Illumina flow cell for cluster generation. Libraries were sequenced on the Genome Analyzer following the manufacturer's protocols.

### Transcriptomic analysis

The transcriptome analysis was performed using Version 1.2.1 of the *P*. *patens* genome and filtered cleaned gene models, which were obtained from http://www.cosmoss.org/. The Illumina reads were mapped to the *P*. *patens* genome using the program TopHat [24, 25]. When mapping the reads to the *P*. *patens* genome, TopHat was run using default settings and a list of existing potential exon junctions was provided. Reads that mapped to unique positions during the first stage of the TopHat mapping process were used to identify stretches of continuous read coverage. This resulted in 309,118 blocks of reads. Next adjacent blocks were then merged if they were within 500bps of each other. After the initial merging any remaining blocks that contained only 1 read were deleted. This resulted in 30,976 blocks of reads. When sequence blocks were joined using TopHat putative splice junctions, a total of 75,461 possible splice junctions were predicted. Only junctions separated by less than 10kbps were used, a cut-off resulting in 99.94% of the junctions being used and only 45 junctions were ignored. This resulted in 29,303 blocks of reads. Next the available gene models were used to further refine the blocks in two ways: (i) if two blocks were within the same gene model then the blocks were joined; (ii) if a block crossed two of the current gene models then it was split into two parts at the midpoint between the two gene models. This resulted in 28,802 sequence blocks, of which 20,347 overlapped with the existing gene models. For manual curation of the alignment, uniquely mapping reads were converted into binary format using SAMtools [26] and then visualised using the Integrative Genomic Viewer (http://www.broadinstitute.org/igv).

Differential expression values for sequence blocks were calculated by counting the number of reads that were present within each block from each sample. These were then divided by the number of uniquely mapping reads (divided by 1 million) present in the sample. This gave an expression for every block from each sample as the number of reads per million reads in the sample. The log-fold change was then calculated and the statistical significance of this change was assessed using a Bayesian method developed for digital gene expression profiling and the assignment of P-values [27], which were corrected for multiple testing [28]. Manual curation and annotation of gene models used BLASTP similarity and conserved domain searches using NCBI conserved domain and SMART (http://smart.embl.de/). Putative signal and transit peptides were identified using TargetP (www.cbs.dtu.dk/services/TargetP).

## Results

### Bleomycin sensitivity

In order to determine a level of DNA damage that would result in the most effective transcriptional induction of DNA-DSB repair components, we first established the sensitivity and responses of *Physcomitrella* protonemal tissue to treatment with the radiomimetic agent, bleomycin. Bleomycin induces DNA-DSBs at high frequency, and *Physcomitrella* is highly sensitive to this drug [29]. We first determined the sensitivity of protonemata to chronic exposure to bleomycin by transferring protonemal explants as a series of “spot inocula” to medium supplemented with a range of bleomycin concentrations.

**Fig 1:**
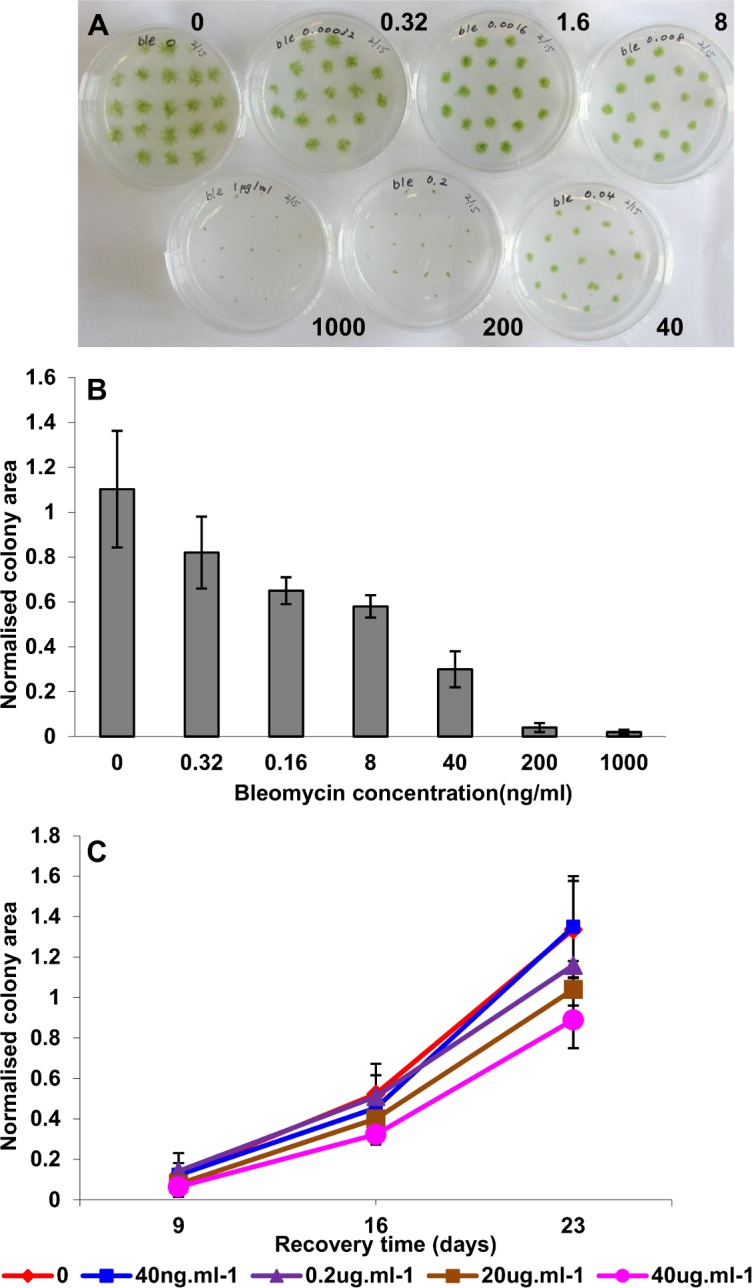
Growth responses of *P*. *patens* to treatment with bleomycin. **(A) Chronic exposure.** Tissue explants were inoculated on BCDAT medium supplemented with bleomycin at 0, 0.32, 1.6, 8, 40, 200 and 1000 ng.ml^-1^ bleomycin as indicated, and incubated for 10 days under standard growth conditions. **(B) Chronic exposure.** Colony areas (mean ± SD) for plants exposed to bleomycin. Colony areas in the plates illustrated in Fig 1a were determined by image analysis of digital photographs of the individual plates, and are presented as the mean colony areas normalized to the area of the culture dish for each treatment. **(C) Acute treatment.** Tissue was incubated for 24h in BCDAT liquid medium supplemented with bleomycin at the concentrations indicated. Explants were then inoculated on drug-free medium and incubated under standard growth conditions for 23d and photographed at 9, 16 and 23d for image analysis. Mean colony areas (± SD, n = 12) were normalized to the area of the culture dish.

Bleomycin treatment caused impaired growth in a concentration-dependent manner, lethality occurring at bleomycin concentrations of 200ng.ml^-1^ or greater (Fig 1A and 1B). Since we sought to establish the parameters for the induction of the DNA damage-response leading to recovery, we then assessed the effects of acute treatment with the drug. Protonemal tissue was incubated in liquid medium containing a range of bleomycin concentrations for 24 hours before explants were subcultured on drug-free medium in order to determine the ability of tissue to recover. Colony growth was monitored at intervals over a 3-week period. Whilst the initial treatment was not lethal, even with relatively high concentrations of bleomycin (40μg.ml^-1^), the recovery of protonemal growth showed a clear concentration-dependence, with the first sign of impaired growth becoming apparent at a concentration of 200ng.ml^-1^ (Fig 1C).

### Transcriptional induction of HR-mediated repair genes

**Fig 2:**
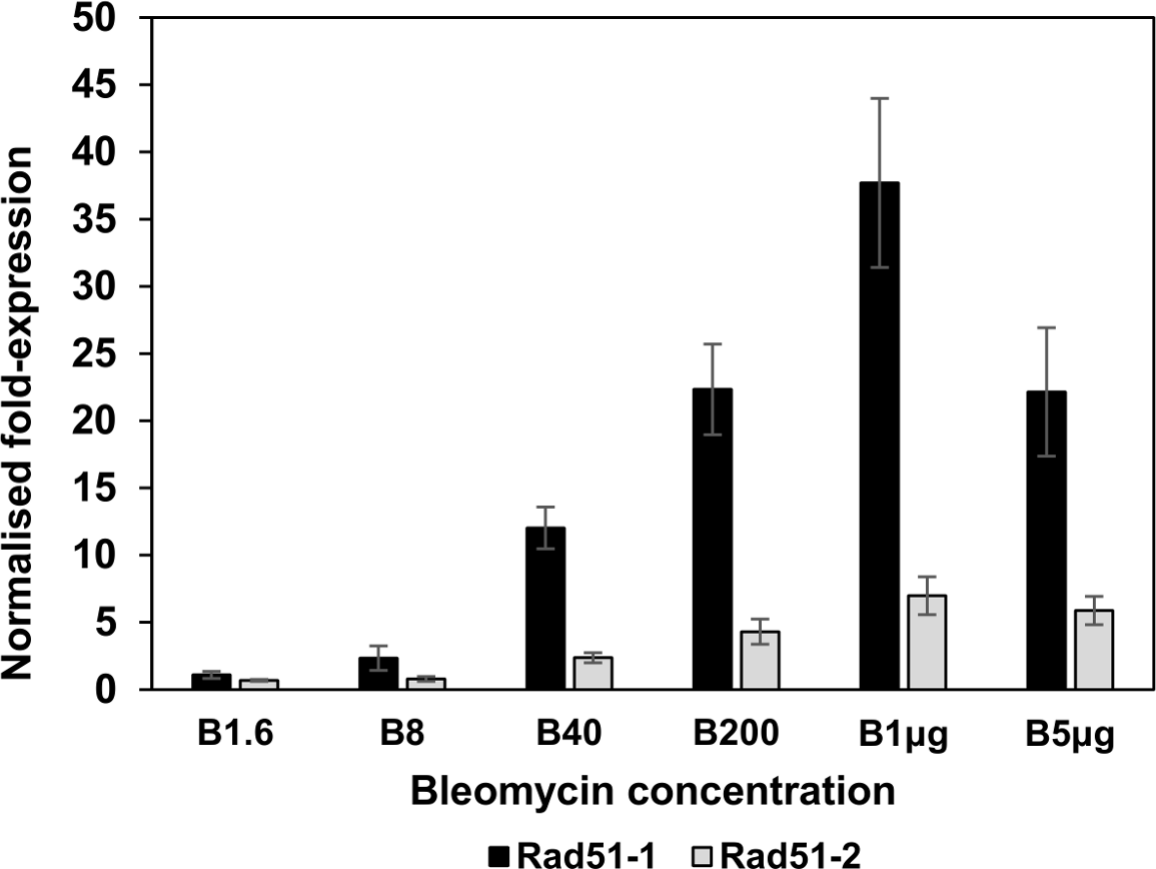
Transcriptional responses of *PpRad51* genes to bleomycin treatment. Tissue was incubated for 24 hours in BCDAT liquid medium supplemented with bleomycin at concentrations of 1.6, 8, 40 and 200ng.ml^-1^ and 1 and 5 μg.ml^-1^ respectively, as indicated. RNA was extracted for determination of transcript levels by real-time qPCR. The fold changes in transcript abundance relative to control (drug-free) treatments are shown (means ± SEM). Black bars: Rad51-1 mRNA; Grey bars: Rad51-2 mRNA.

We next monitored HR-mediated DNA repair gene expression following induction of DNA-DSBs. Protonemal tissue was incubated in liquid medium containing a sublethal concentration of bleomycin for 24 hours, after which accumulation of the two *Physcomitrella* Rad51 transcripts was measured by quantitative real-time PCR. Since the Rad51 proteins are key components of the HR-mediated DNA-DSB repair process, and absolutely required for gene targeting in *Physcomitrella* [21], induction of their transcripts is indicative of the transcriptional induction of this pathway, which would additionally be expected to encompass other components required for HR-mediated DNA repair and transgene integration. Both Rad51 transcripts were induced by bleomycin treatment, with expression of the *RAD51-1* gene being significantly more strongly induced than that of the *RAD51-2* gene, whose transcript level remained relatively low (Fig 2). Accumulation of the Rad51-1 transcript showed a clear correlation between the level of accumulation and the concentration of bleomycin (and by extension, the extent of DNA-DSB induction) peaking at the previously determined sublethal acute doses of 200ng.ml^-1^ and 1μg.ml^-1^.

### Transcriptomic analysis

We incubated protonemal tissue in the presence or absence of 200ng.ml^-1^ bleomycin for 24 hours, before isolating a polyribosome-enriched fraction from triplicate samples for the extraction of RNA actively engaged in translation. Each sample was analysed by real-time PCR for the *PpRAD51-1* transcript, which was found to be reproducibly enhanced approx. 30-fold (30.4 ± 3.8) in each bleomycin-treated sample. For transcriptomic analysis, equal quantities of the three control (untreated) RNA samples were pooled, as were the three bleomycin-treated RNA samples, in order to further minimise variation between replicates before being submitted to cDNA synthesis and Illumina short-read sequence analysis.

A total of 30,487,204 Illumina short reads (40 bases) were obtained, corresponding to a total of 1,219,488,160 bases. Of these, 15,942,360 reads were obtained from the control treatment and 14,544,845 from the bleomycin treatment. In total, 21,399,712 of the reads (70%) were mapped to the *Physcomitrella* genome assembly, Version 1.2.1 obtained from http://www.cosmoss.org, with 20,931,284 (69%) of them mapping to unique positions (11,183,882 from the control treatment and 9,747,402 from the bleomycin treatment). Assembly of reads into contiguous blocks, aligned with the genome assembly generated 28,802 sequence blocks, of which 20,347 overlapped with the Version 1.2.1 filtered gene models. From this, we estimate that approximately two-thirds of *P*. *patens* genes are expressed in chloronemal filaments. These alignments have been integrated into the Cosmoss genome browser, where they have been used in the generation of the Version 1.6 and 3.3 gene models, and are available for further structural gene annotation.

To identify genes up-regulated in response to the induction of DNA-DSBs, we used an automated procedure for digital gene expression analysis. The entire dataset, filtered by Log fold-change and P-value, has been deposited in the GEO database (http://www.ncbi.nlm.nih.gov/geo) as Accession GSE25237. We undertook a detailed manual curation of those genes identified as significantly up- and down-regulated in response to bleomycin. In order to make such a task manageable, candidates were limited (i) to those genes exhibiting a change in expression level of 3-fold or greater and (ii) applying a cutoff based on selecting only those genes represented by at least 50 sequence traces in one or the other treatment, to exclude stochastic variation. This latter criterion excludes a significant number of genes expressed at a low level (approx. 40% of all genes identified in the experiment), but still provided a list of 500 up-regulated and 380 down-regulated sequences. These genes are listed in the Supporting Information files (S1 Dataset: up-regulated genes and S2 Dataset: down-regulated genes). Because the *Physcomitrella* genome has been undergoing continual refinement and reassembly, the list is annotated with a range of accession numbers (Locus identifiers, protein IDs) used in the successive assemblies.

### Verification of transcriptomic analysis

**Fig 3:**
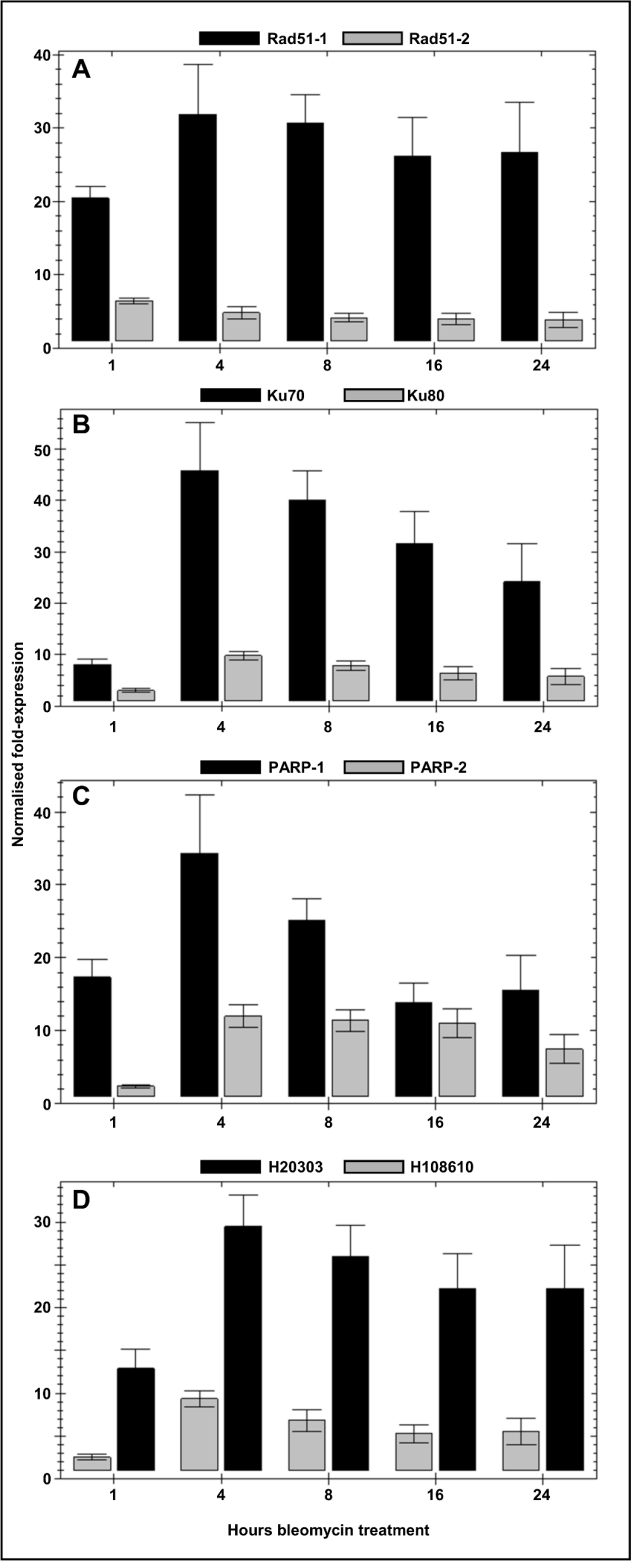
Time-course of the transcriptional response of selected DNA repair genes. Tissue was incubated in BCDAT medium supplemented with 200ng.ml^-1^ for 1, 4, 8, 16 and 24 hours prior to harvest. RNA was extracted for determination of transcript levels by real-time qPCR. The fold changes in transcript abundance relative to control (drug-free) treatments are shown (means ± SEM). **(A)** Rad51-1 mRNA (black bars); Rad51-2 mRNA (grey bars). **(B)** Ku70 (black bars); Ku80 (grey bars). **(C)** PARP-1 (Pp3c22_13240V3.1) (black bars); PARP-2 (Pp3c8_17220V3.1) (grey bars); **(D)** SRS2-like helicase (Pp3c1_29170V3.1) (black bars); Alc1-like helicase (Pp3c10_6710V3.1) (grey bars).

We used real-time PCR to verify the results of the global transcriptome analysis, by monitoring the time-course of accumulation of selected transcripts in response to 200ng.ml^-1^ bleomycin. Target genes were selected from among those identified as significantly up-regulated in the transcriptome-wide analysis. As exemplars of the HR-mediated DNA-DSB repair pathway, we analysed the Rad51-1 and Rad51-2 transcripts (Fig 3A), for the NHEJ pathway, we selected the Ku70 and Ku80 transcripts (Fig 3B). We also selected two genes encoding polyADP ribose polymerase (PARP) (Fig 3C) and two genes encoding DNA helicases (Fig 3D). In each case, a general trend is clear. First, gene induction is rapid: significant accumulation of each transcript relative to that in the control treatment is detectable after 1 hour of bleomycin treatment, with a peak of expression seen at the 4-hour time-point. Second, although the levels of gene induction relative to the control treatments at the 24-hour time-point do not exactly replicate those determined in the transcriptome analysis for each gene, the trends are generally similar. Third, for each category of gene analysed by real-time PCR, the levels of induction of the selected gene pairs relative to each other is similar to that identified in the transcriptomic analysis. We are thus confident that the genome-wide assay of gene induction provides a true reflection of the protonemal response to bleomycin-induced DNA damage.

### Functional analysis of selected genes

The catalogue of genes significantly induced in response to DNA damage includes those implicated in all DNA repair pathways, not only the HR-mediated repair pathway that is responsible for mediating high-efficiency gene targeting in *P*. *patens*. Table 1 lists the up-regulated genes that function in DNA-DSB repair by the NHEJ, and MMEJ pathways, as well as components associated with nucleotide-excision repair and base-excision repair.

**Table 1:** Up-regulated DNA repair and replication genes.

Fold change	V3.3 ID	Phypa1_1:ID and annotation
149x	Pp3c1_38430V3.1	86560: NHEJ DNA-repair protein XRCC4
52x	Pp3c22_13240V3.1	150949 NAD^+^ ADP-ribosyltransferase PARP-1
27x	Pp3c16_1250V3.1	190334: HhH-GPD base-excision repair family protein
21x	Pp3c8_19560V3.1	166702: Chromosome segregation ATPase
20x	Pp3c24_12060V3.1	20647: minichromosome maintenance family protein 8
19x	Pp3c14_5920V3.1	228728: NHEJ-associated DNA ligase 4
15x	Pp3c13_17960V3.1	86100: Fanconi anemia, complementation group I gene involved in interstrand crosslink repair.
14x	Pp3c2_30860V3.1	200726: UV DNA repair protein: Flap endonuclease 1a
14x	Pp3c18_7140V3.1	60909: PpKu70 NHEJ DNA repair protein
13x	Pp3c12_3560V3.1	173451: Similar to telomere maintenance component 1
11x	Pp3c6_22830V3.1	82469: SMC class chromosome segregation protein
9x	Pp3c6_17700V3.1	203240: HhH-GPD base excision DNA repair family protein
8x	Pp3c13_20209V3.1	40610: DNA ligase 1
8x	Pp3c11_22000V3.1	206066 HR DNA repair PpRad51-1
8x	Pp3c4_21450V3.1	171541: DNA polymerase delta, subunit 4
8x	Pp3c16_2140V3.1	235804: PpCtIP (yeast Sae2) HR End resection nuclease
7x	Pp3c6_3460V3.1	129584: PpATR HR checkpoint activation kinase
7x	Pp3c5_18670V3.1	226781: Metallo-beta-lactamase (β-CASP) interstrand DNA cross-link pair protein-related
7x	Pp3c22_7360V3.1	115005: Rad4-like nucleotide excision repair protein
7x	Pp3c18_11670V3.1	201653: ScRAD1 DNA repair endonuclease (hsERCC4)
6x	Pp3c8_21590V3.1	231531: Topoisomerase-interacting protein
6x	Pp3c10_1920V3.1	93627: Excinucleases ABC, C subunit protein
6x	Pp3c8_17250V3.1	134789: UMUC-like DNA-repair protein: Rad30
6x	Pp3c11_21910V3.1	161308: Sister chromatid cohesion protein (cohesin)
6x	Pp3c17_7580V3.1	95275: HMG1/2 protein (high mobility group b2)
5x	Pp3c17_18800V3.1	164103: DNA polymerase B: delta subunit
5x	Pp3c4_1630V3.1	167899: C-terminal BRCT domain–containing protein
5x	Pp3c8_17220V3.1	188096: poly(ADP-ribose) polymerase (PARP-2)
5x	Pp3c27_130V3.1	148594: Nse4: Smc5/6 DNA repair complex member
5x	Pp3c8_16660V3.1	188047: "damaged DNA -binding protein 2" (DDB2)
5x	Pp3c22_11100V3.1	188592: PpKu80 NHEJ DNA repair protein.
4x	Pp3c2_33440V3.1	147728: DNA polymerase B zeta subunit
4x	Pp3c20_10450V3.1	11886: Replication protein A subunit 1: PpRPA-1a
4x	Pp3c23_16880V3.1	144964: Replication Protein A subunit 1: PpRPA-1b
3x	Pp3c22_21030V3.1	145270: DNA replication factor C complex subunit 1
3x	Pp3c14_15380V3.1	107931: Uracil DNA glycosidase

We tested the contributions made by some of these genes to the DNA-damage response by constructing targeted knockout mutants. Genes were selected either because their homologues had been implicated in DNA-DSB repair in other organisms, or because they appeared functionally interesting. As a gene known to be important for homology-dependent repair, encoding the initiator of end-resection that characterises this pathway, we generated a targeted deletion of *PpCtIP* (Pp3c16_2140V3.1) (S1 Fig), encoding the orthologue of the human and yeast genes, *CtIP* and *SAE2*, respectively [30–32]. Additionally, we noted that a small group of chromatin-remodelling DNA helicases were represented among the strongly damage-induced genes (Table 2). These included *PpTEBICHI*, (Pp3c5_12930V3.1) (S2 Fig)–a helicase with a DNA polymerase theta domain implicated in replication-related MMEJ [33, 34], a helicase/nuclease *PpZRL* (for “ZRANB3-Like”–a member of the SMARCA L1 Snf2 helicase subfamily, Pp3c1_13308V3.1) (S3 Fig), *PpALC1* (Pp3c10_6710V3.1) (S4 Fig) a homologue of human “Amplified in Liver Cancer 1” [35], a chromodomain-helicase gene *PpCHD5* (Pp3c20_11500V3.1) (S5 Fig) similar to the *Arabidopsis* helicase AtCHR5 and the developmental regulator, *PICKLE* [36, 37], the Rad3-like gene *PpRTEL1* “Regulator of telomere elongation”: Pp3c18_12550V3.1) (S6 Fig), *PpSRS2* (Pp3c1_29170V3.1) and *pERCC6* (Pp3c15_23590V3.1) (S7 Fig). Significantly, among the Arabidopsis orthologues of these genes, the ERCC6 (AT2G18760) gene was the only one to be significantly up-regulated in response to DNA damage [38], suggesting that the differential inducibility of these genes by DNA damage might reflect the relative competences to undertake targeted transgene integration between moss and angiosperms.

**Table 2:** Up-regulated genes annotated as DNA helicases.

Fold change	V3.3 ID	Arabidopsisorthologue	Fold change (Arabidopsis)	Functional annotation
25x	Pp3c1_29170V3.1	AT4G25120	1.4x down	ATP-dependent DNA helicase similar to yeast SRS2 *PpSRS2*
16x	Pp3c10_6710V3.1	AT2G44980	1.1x down	Alc1-like SNF2 family DNA- dependent ATPase/helicase
13x	Pp3c8_17910V3.1	none	-	SNF2 family DNA-dependent ATPase/helicase Rad54B
11x	Pp3c15_23590V3.1	AT2G18760	6.7x up	ERCC6-like SNF2 helicase *PpERCC6*
11x	Pp3c5_12930V3.1	At4G32700	1.1x down	Tebichi ortholog: polymerase with DEAD/DEAH box and polymerase theta helicase domains *PpTEB*
11x	Pp3c1_13308V3.1	AT5G07810	1.4x down	SNF2-related DNA Helicase/endonuclease *PpZRL*
8x	Pp3c20_11500V3.1	AT2G13370	1.2x down	SNF2 family chromodomain-helicase *PpCHD5*
6x	Pp3c18_12550V3.1	AT1G79950	No change (1x)	RTEL1-like helicase *PpRTEL*
5x	Pp3c10_16570V3.1	AT1G03190	1.2x down	ERCC2-like helicase *PpERCC2*

Up-regulated *P*. *patens* helicases compared with the DNA-damage (100Gy γ-irradiation) induced changes in expression of their orthologues in wild-type *A*. *thaliana*, as reported by Culligan *et al*. [38].

All mutants subsequently used for phenotypic analysis were verified as being correctly targeted gene replacements, in which the native gene was replaced by a single copy of the gene disruption cassette (S1–S7 Figs). The mutant lines were analyzed in two ways. First, by determining whether they displayed hypersensitivity to DNA damage caused by the genotoxin bleomycin, and secondly by analyzing the efficiency with which targeted transgene integration occurred following protoplast transfection.

**Fig 4:**
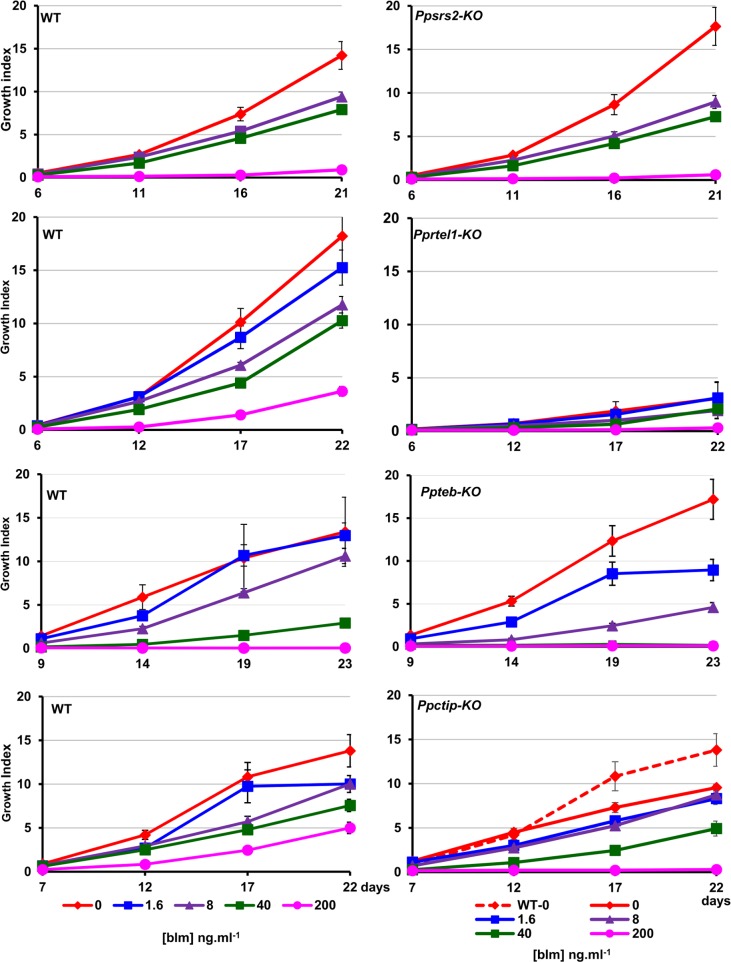
Bleomycin sensitivity of *P*. *patens* mutants. Tissue explants of wild-type and mutant protonemal tissue were inoculated on BCDAT agar medium, supplemented with a 5-fold dilution series of bleomycin (1.6ng.ml^-1^ – 200ng.ml^-1^). Plants were grown for a period of approximately 3 weeks, and plant growth was determined by image analysis of digital photographs taken at intervals to generate the Growth Index. WT and mutant plants (8–12 explants) were inoculated on the same plates for direct comparison. Because individual experiments took place at intervals over a period of 2 years, direct comparisons cannot be made between each pair of panels, as different batches of bleomycin with varying potency were used during this time. Mutants analysed (from top) were *Ppsrs2-KO; Pprtel1-KO* (note the 5-fold difference in the amplitudes of the y-axes); *Ppteb-KO* and *Pctip-KO*. Representative images of plant growth are shown in S9 Fig.

In growth tests, the *Ppercc6*, *Ppzrl*, *Ppchd5*, and *Ppalc1* mutant lines displayed no discernible growth defects or hypersensitivity to DNA damage (S8 Fig). Neither did the *Ppsrs2* mutant, although the *Pprtel1* mutant exhibited a severe growth defect, plants achieving only one-sixth of the size of the corresponding wild-type. However, this mutant showed no discernible hypersensitivity to genotoxin (Fig 4; S9 Fig). By contrast, the *Ppctip* mutants grew slightly less rapidly under normal conditions, and were sensitised to DNA damage, being susceptible to 200ng.ml^-1^ bleomycin, whereas the wild-type plants survived this dose. The *Ppteb* mutant plants also exhibited a similar hypersensitivity to bleomycin, but showed no growth defects on unsupplemented growth medium (Fig 4; S9 Fig).

These mutants were also tested for their ability to undertake targeted transgene integration (with the exception of the *Pprtel* mutant line, which grew too slowly for the isolation of protoplasts suitable for transformation). Only the *Ppctip* mutant exhibited a clear severe gene targeting deficiency (Table 3), a finding consistent with the conserved role of the CtIP/Sae function in initiating resection of double-strand breaks during homology-dependent repair. The *Ppchd5*, *Ppzrl* and *Ppercc6* all undertook targeted integration at a frequency some 5–10% below that of the wild-type, but this is not thought to represent a significant difference between the lines. The *Ppalc1* and *Ppteb* mutants showed no defects in transformation efficiency or transgene integration.

**Table 3:** Gene targeting efficiency in mutant lines.

Strain	RTF (%)	Hyg^R^	2-FA^R^	%GT
WT	1.14	1183	843	73.3% (72.1 ± 5.2: n = 3)
*Ppalc1-KO*	n.d.	506	397	78.4% (79.3 ± 5.6: n = 3)
*Ppchd5-KO*	1.77	1600	1081	67.6% (67.6 ± 7.9: n = 3)
*Ppzrl-KO*	0.68	345	220	63.7% (65 ± 11.2: n = 3)
*Ppteb-KO*	1.65	1149	910	79.2% (79.3 ± 3.9: n = 3)
*Ppctip-KO*	n.d.	285	64	22.5% (22.2 ± 4.7: n = 6)
*Ppercc6-KO*	2.87	2680	1739	64.9% (61.7 ± 12.8: n = 3)
*Ppsrs2-KO*	1.33	1266	923	72.9% (72 ± 3: n = 3)

RTF (Relative transformation frequency) is the proportion of viable protoplasts that regenerate into hygromycin-resistant plants following transformation. Hyg^R^ (hygromycin resistant) is the number of stably transformed plants containing the *APT* targeting construct. 2-FA^R^ (2-fluoroadenine resistant) is the number of plants resistant to 2-fluoroadenine. %GT (% gene targeting) is the proportion of stable transformants that are resistant to 2-fluoroadenine: (2-FA^R^/Hyg^R^)x100.

n.d.: Not determined.

PpSRS2 is a homologue of the yeast SRS2 helicase, often termed an “anti-recombinase”, which interacts with and is activated by Rad51 in HR-mediated DNA repair. It is posited to be important in preventing unwanted gene conversion events in homology-dependent repair of DSBs through disassembling Rad51-ssDNA complexes [39, 40]. We have previously demonstrated that targeted knockout of *PpSRS2* has no phenotypic effects in *Physcomitrella*, either for DNA damage-sensitivity or gene targeting efficiency, and neither did its deletion relieve a GT deficiency resulting from the knockout of the RAD51 paralogue RAD51B [41]. We therefore analyzed whether deletion of this gene would render *P*. *patens* susceptible to targeted integration of mismatched transgenes, by analyzing the integration of a hygromycin resistance marker into the *PpAPT* locus by targeting sequences containing a 1%, 2% or 3% complement of mismatches [42]. Unlike the *msh2* mutation, deficient in mismatch repair [42], the *srs2-KO* mutant did not enhance the targeted integration of mismatched sequences (Table 4). The frequencies of targeted transgene integration obtained with these constructs were essentially identical to those previously observed in wild-type *P*. *patens* [42], and the relative transformation frequency obtained with these constructs showed a substantial decrease, proportional to the extent of mismatch, demonstrating the very strong preference for the integration of transforming DNA at homologous sites over illegitimate recombination [5, 16, 21].

**Table 4:** Gene targeting by *Ppapt-KO* mismatch vectors in *Ppsrs2-KO*.

Strain	Mismatch	Hyg^R^	2-FA^R^	%GT	RTF(%)
*Ppsrs2-KO*	0% mismatch	248	52	41.9*	1.12
*Ppsrs2-KO*	1% mismatch	48	13	16.7	0.10
*Ppsrs2-KO*	2% mismatch	17	5	5.9	0.10
*Ppsrs2-KO*	3% mismatch	26	8	0	0.006

*GT efficiencies vary, depending on the nature of the targeting construct (Kamisugi *et al*., 2005). Thus the GT frequency obtained with this vector cannot be compared with that obtained with the different *PpAPT* targeting vector used for the experiments reported in Table 3.

## Discussion

Bleomycin induces changes in gene expression of not only DNA-repair components but a range of other functions reflecting a general stress-response (S1 Dataset and S2 Dataset).

### DNA repair components

Several up-regulated DNA-repair genes (Table 1) have a highly conserved role in HR-mediated repair [43]. These include the PpRAD51-1 recombinase, the ATR kinase that executes a cell-cycle checkpoint at G_2_/M to facilitate HR-mediated DNA-DSB repair [12] and that co-activates the up-regulated end-resection protein CtIP (Sae2 in yeast) [30–32, 44, 45] and both genes encoding the largest subunit of the single-strand DNA-binding Replication Protein A complex. Unsurprisingly, targeted knockout of *PpCtIP* resulted in enhanced sensitivity to genotoxic stress and a significant reduction in GT efficiency–although GT was not abolished as occurs following deletion of *RAD51* paralogues [21, 41].

Other DNA repair functions are also represented. The RAD1 exonuclease (with RAD10 and the MSH2/3 mismatch repair system) trims non-homologous sequences from the ends of transforming DNA prior to insertion by HR in yeast [46], and is implicated in plant somatic recombination [47]. Two polyADP-ribose polymerase (PARP) genes are strongly induced. PARPs participate in a range of DNA repair activities, including MMEJ and base-excision repair, and transcriptional activation of PARP genes by genotoxic stress is characteristic of mammalian cells [48]. In *Physcomitrella*, two PARP genes (*PARP-1*: Pp3c22_13240V3.1 and *PARP-2*: Pp3c8_17220V3.1) are strongly up-regulated. The third (*PARP-3*: Pp3c1_22640V3.1) is not significantly expressed, being represented by only a single sequence read. *PpTEBICHI* encodes a helicase-DNA polymerase theta, an enzyme that in metazoans processes MMEJ ends [49]. In *P*. *patens*, MMEJ has been implicated in transgene concatenation prior to “one-ended” targeted insertion into the genome [50], a process analogous to the targeted integration of multiple tandem transgenes in yeast [51]. The *Ppteb-KO* mutant showed enhanced bleomycin sensitivity, but no recombination defect or serious developmental phenotype. By contrast, the corresponding *teb* mutant in *Arabidopsis* displays constitutively activated DNA-damage responses, an inability of meristematic cells to progress through the G_2_/M transition, and a deficiency in intrachromosomal recombination [33, 34]. This contrast likely reflects the greater prominence of the HR repair pathway in *P*. *patens*, compared with *A*. *thaliana*.

Up-regulated NHEJ-related genes include Ku70, Ku80, XRCC4 and DNA ligase 4. Interestingly several of these are up-regulated to a greater extent than are HR-related genes. This may reflect a preference for the HR pathway in normal growth and development of *P*. *patens*, such that NHEJ-associated gene products are generally maintained at a relatively low constitutive cellular concentration, and are only required to be produced in response to the induction of extensive DNA damage.

### Cell-cycle regulation

Cell-cycle checkpoint imposition is a critical part of the DNA damage response. Differentially regulated genes associated with cell-cycle checkpoint control include *PpATR* and homologues of the *S*. *pombe RAD9* and *HUS1* genes whose products combine (with that of *SpRad1*, in the “9-1-1” complex) to execute a G_2_/M checkpoint [52]. The down-regulated gene set includes both D-type cyclin and B-type cyclin-dependent kinase genes required for cell-cycle progression (Table 5).

**Table 5:** Differentially regulated genes annotated as cell-cycle associatedFold change.

	V3.3 ID	Phypa1_1:ID and annotation
**Up-regulated**		
50x	Pp3c7_570V3.1	234322: Checkpoint 9-1-1 complex, RAD9 component
24x	Pp3c21_200V3.1	197198: AAA-type ATPase (cell-division related)
11x	Pp3c1_35610V3.1	160969: AAA-ATPase
8x	Pp3c10_18160V3.1	219794: cdc20 anaphase promoting complex protein
4x	Pp3c1_24880V3.1	106047: Cell cycle checkpoint protein Hus1
3x	Pp3c9_2440V3.1	10529: Retinoblastoma-related protein
**Down-regulated**		
5x	Pp3c15_17470V3.1	226408: Cyclin D
5x	Pp3c16_3910V3.1	107101: B-type cyclin-dependent kinase
6x	Pp3c15_21520V3.1	196659: Cyclin B
44x	Pp3c27_6070V3.1	60060: B-type cyclin-dependent kinase cdc2

### Housekeeping genes

Many housekeeping functions are downregulated (S2 Dataset), in particular protein synthesis, photosynthesis and cell growth: 186 of the downregulated genes encode ribosomal proteins, probably reflecting a checkpoint-mediated reduction in mitotic activity with which the synthesis and assembly of ribosomes are closely correlated. Down-regulation of photosynthetic functions is likely a general abiotic stress response, since loss of photosynthetic function is one of the most striking features of salt- and drought-stress in *Physcomitrella* [53]. However, the responses to bleomycin-induced DNA damage are distinct from those associated with drought stress, in that a number of characteristically drought-responsive genes [53, 54] are down-regulated following bleomycin treatment (S3 Table).

Reduction of mitotic activity and cell elongation, would explain the reduced growth rate seen in response to bleomycin treatment, and a number of genes with growth-regulating cell wall-modifying properties are also represented in both the up- and down-regulated gene sets. These include expansins [55], xyloglucan endotransglycosidases [56], pectin modifying enzymes, arabinogalactan proteins and germin-like proteins.

### Transcription factors

Bleomycin treatment induces significant changes in the expression profiles of transcription factors that implement essential cellular damage control measures. Thirty-six transcription factor genes appear in the up-regulated set and fourteen in the down-regulated set (S4 Table and S5 Table).

Members of the AP2/EREBP-domain family are most prominent (16 up-regulated and 4 down-regulated). A highly expanded plant transcription factor family, this comprises nearly 150 members in *Arabidopsis* [57] and a similar number (approx. 135) in *Physcomitrella*, so these might be expected to comprise a significant fraction of the differentially regulated transcription factor subset. Additionally, many AP2/EREBP transcription factors regulate responses to both biotic and abiotic stresses that involve the intracellular generation of active oxygen species (AOS) (pathogen attack, drought, wounding), and their induction in response to bleomycin may reflect secondary effects of AOS generated by the drug’s mode of action. Notably, genes whose products ameliorate the cytotoxic consequences of AOS are also up-regulated, including lipases (14 up-regulated) and three tetratricopeptide-repeat containing thioredoxins, as well as genes known to be responsive to AOS in pathogen responses, such as the 8 chalcone synthase genes and 3 phenylalanine ammonia lyase genes identified in this study. These enzymes are components of the phenylpropanoid biosynthetic pathway, regulated by R2R3 myb-family transcription factors [58–60] and we note that two such transcription factor genes are highly up-regulated.

### Candidate modulators of gene targeting

A rationale for this study was to determine whether genes up-regulated in response to DNA-DSB induction were associated with the high frequency of HR-mediated GT. We therefore compared the DNA-damage transcriptomes of GT-competent *Physcomitrella* and GT-incompetent *Arabidopsis* to identify potential candidates. Several genome-wide studies of the *Arabidopsis* DNA-DSB response have been carried out, utilising macro- and microarray platforms to identify genes responsive to genotoxic chemicals (bleomycin, mitomycin C and methane methylsulphonate: [61, 62] and to ionising radiation (X- and γ-irradiation, [38, 63].

We identified a number of up-regulated moss-specific genes encoding chromatin remodeling DNA helicase functions (Table 2) and selected these for further analysis since DNA helicases of the Swi2/Snf2 family are implicated in intrachromosomal recombination between inverted repeats in *Arabidopsis* [37], and different helicases are associated with the two different mechanisms of HR-mediated DSB repair: the single-strand annealing mechanism and the synthesis-dependent strand annealing pathway [64].

The Alc1 helicase relocates within mammalian nuclei to the site of DNA-DSBs within seconds of their induction [35] in a process involving activation of its ATPase activity by PARylation by nucleosome-bound PARP to mediate DNA repair [65]. Since two PARP genes were also upregulated by bleomycin, this suggested a potential role in DNA-DSB repair and gene targeting. In mammalian cells, both overexpression and RNA-mediated knock-down of *ALC1* result in sensitivity to genotoxins. By contrast, knockout of *ALC1* appeared neither to compromise growth of *P*. *patens* nor affect its ability to undertake targeted transgene integration.

Shaked *et al*. [37] reported that *Arabidopsis* mutants of the *PpERCC6* orthologue, At2g18760, exhibited sensitivity to UV-C but not to γ-irradiation: a response consistent with its predicted role in nucleotide excision repair. We found the *Ppercc6-KO* mutant to show no growth deficiency or bleomycin sensitivity, and although GT efficiency in the mutant was lower than that in the wild-type, it is doubtful that this is a significant difference. Alone among the genes we tested, *PpERCC6* is represented by additional paralogues in the *P*. *patens* genome (there are 3 other closely related family members, none of which were up-regulated following bleomycin treatment), so genetic redundancy likely accounts for the lack of a clearly observable deleterious phenotype.

By contrast, knockout of the *PpRTEL1* orthologue generated a clear, general growth deficiency. However this was unrelated to the presence or absence of bleomycin, and most likely represents a deficiency in telomere maintenance: mutations of this gene in different organisms consistently result in severe phenotypic consequences, such as the human degenerative condition Dyskeratosis congenita. In *Arabidopsis*, mutation of *AtRTEL1* has severe pleiotropic effects that include growth retardation, suppressed homologous recombination and defects in both intra- and interstrand crosslink repair [66] and cell-cycle arrest [67]. Due to the difficulty of generating viable protoplasts from this mutant, we were unable to determine its impact on gene targeting.

*Physcomitrella patens* is extraordinarily robust in its ability to withstand genotoxic stress. This may be a necessary characteristic for an organism that spends most of its life cycle in the haploid phase. Although it has been postulated that the dominant form of DNA-DSB repair is the homology-dependent pathway, analysis of the DNA-damage transcriptome reveals the rapid induction of genes encoding components active in most forms of DNA repair process, including nucleotide excision repair, base excision repair, transcription coupled repair, inter- and intra-strand crosslink repair, NHEJ- and HR-mediated DNA-DSB repair and in DNA-damage associated checkpoint signalling. Targeted knockout of a number of individual genes identified in this analysis had surprisingly little adverse effect on either growth, sensitivity to genotoxin or to targeted transgene integration, unlike the consequences of their mutation in other organisms, suggesting a high level of built-in redundancy in genome maintenance.

## Supporting Information

S1 Dataset Genes up-regulated by bleomycin. (XLSX)

S2 Dataset Genes down-regulated by bleomycin. (XLSX)

S1 Fig PpCtIP-KO. **A:** Schematic of gene structure and knockout construct. **B:** Identification of targeted loci by PCR amplification with cassette-specific “outward” and gene-specific “inward” primers **C:** Identification of single-copy targeted transformants with external gene-specific primers **D:** Southern blot (*Hin*dIII digest) to identify transformants containing only a single, targeted selection cassette. In S1–S7 Figs, Red boxes = protein-coding sequence, Blue boxes = 5’- and 3’-UTRs, Green boxes = *NPTII* selection cassette, Red triangles = *LoxP* sites. In Southern blots, the *NPTII* sequence was used as a probe.
(PDF)

S2 Fig Ppteb-KO. **A:** Schematic of gene structure and knockout construct. **B:** Identification of targeted loci by PCR amplification with cassette-specific “outward” and gene-specific “inward” primers. **C:** Identification of single-copy targeted transformants with external gene-specific primers (track “P” = plasmid control). **D:** Southern blot (*Eco*RI digest) to identify transformants containing only a single, targeted selection cassette.
(PDF)

S3 Fig Ppzrl-KO. **A:** Schematic of gene structure and knockout construct. **B:** Identification of targeted loci by PCR amplification with cassette-specific “outward” and gene-specific “inward” primers. **C:** Identification of single-copy targeted transformants with external gene-specific primers (Track “P” = plasmid control). **D:** Southern blot (*Hin*dIII digest) *to* identify transformants containing only a single, targeted selection cassette. (Analysed on same gel as *PptebKO*: see S2 Fig for WT control).
(PPTX)

S4 Fig Ppalc1-KO. **A:** Schematic of gene structure and knockout construct. **B:** Identification of targeted loci by PCR amplification with cassette-specific “outward” and gene-specific “inward” primers **C:** Identification of single-copy targeted transformants with external gene-specific primers (Tracks “P” and “wt” = plasmid and wild-type genomic DNA controls) **D:** Southern blot (*Hin*dIII digest) to identify transformants containing only a single, targeted selection cassette. Analysed on same gel as *Ppchd5-KO*: see S5 Fig for wild-type control).
(PPTX)

S5 Fig Ppchd5-KO. **A:** Schematic of gene structure and knockout construct. **B:** Identification of targeted loci by PCR amplification with cassette-specific “outward” and gene-specific “inward” primers **C:** Identification of single-copy targeted Transformants with external gene-specific primers (Tracks “P” and “wt” = plasmid and wild-type genomic DNA controls). **D:** Southern blot (*Bgl*II digest) to identify transformants containing only a single, targeted selection cassette.
(PPTX)

S6 Fig Pprtel-KO. **A:** Schematic of gene structure and knockout construct. **B**: Identification of targeted loci by PCR amplification with cassette-specific “outward” and gene-specific “inward” primers. **C:** Identification of single-copy targeted transformants with external gene-specific primers (Track “P” = plasmid control). **D:** Southern blot (*Eco*RV digest) to identify transformants containing only a single, targeted selection cassette.
(PPTX)

S7 Fig Ppercc6-KO. **A:** Schematic of gene structure and knockout construct. **B:** Identification of targeted loci by PCR amplification with cassette-specific “outward” and gene-specific “inward” primers **C:** Identification of single-copy targeted transformants with external gene-specific Primers primers (Tracks “P” and “wt” = plasmid and wild-type genomic DNA controls). **D:** Southern blot (*Bg*lII digest) to identify transformants containing only a single, targeted selection cassette
(PPTX)

S8 Fig Bleomycin sensitivity of moss mutants. (PPTX)

S9 Fig Photographs of WT and mutant plants on control and bleomycin-supplemented medium. Each photograph represents the final time-point presented in the growth test analysis in Fig 4. For *Ppteb-KO* the growth on 40ng.ml^-1^ bleomycin is shown. For all other mutants, growth on 200ng.ml^-1^ bleomycin is shown.
(PPTX)

S1 Table Primers used to construct gene targeting vectors. (PDF)

S2 Table Primers used for real-time quantitative PCR. (PDF)

S3 Table Down-regulated genes that are up-regulated by drought-stress. (PDF)

S4 Table Up-regulated genes annotated as transcription factors. (PDF)

S5 Table Down-regulated genes annotated as transcription factors. (PDF)
