# Supplementary material for: The Transcriptional Response to DNA-Double-Strand Breaks in Physcomitrella patens
Source: PLoS One. 2016 Aug 18;11(8):e0161204. doi: 10.1371/journal.pone.0161204 (PMC4990234; doi:10.1371/journal.pone.0161204)
Supplement: S1 Fig — A: Schematic of gene structure and knockout construct. B: Identification of targeted loci by PCR amplification with cassette-specific “outward” and gene-specific “inward” primers C: Identification of single-copy targeted transformants with external gene-specific primers D: Southern blot (HindIII digest) to identify transformants containing only a single, targeted selection cassette. In S1–S7 Figs, Red boxes = protein-coding sequence, Blue boxes = 5’- and 3’-UTRs, Green boxes = NPTII selection cassette, Red triangles = LoxP sites. In Southern blots, the NPTII sequence was used as a probe. (PDF) [file pone.0161204.s003.pdf]

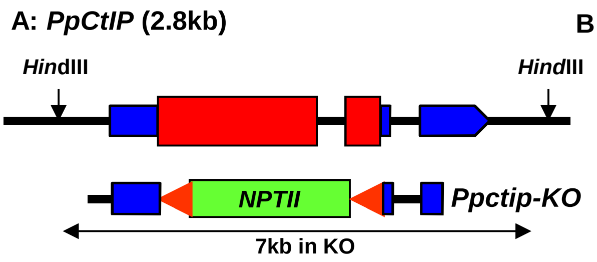

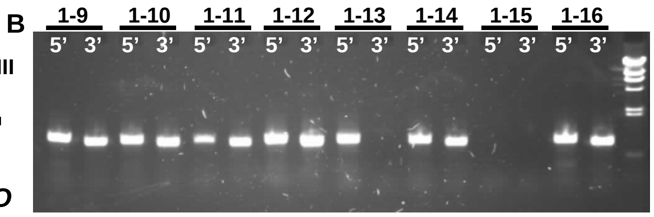

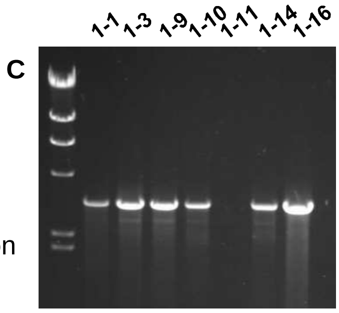

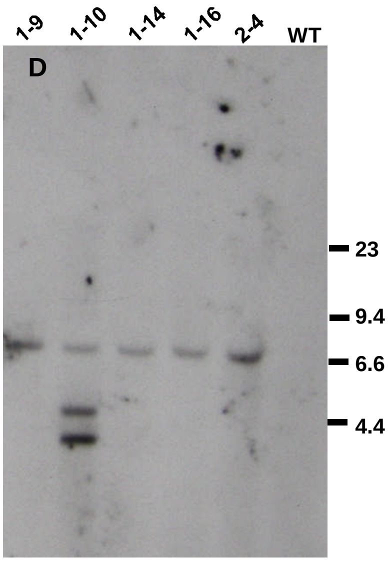

S1 Fig: *Ppctip-KO*
A: Schematic of gene structure and knockout construct.
B: Identification of targeted loci by PCR amplification with cassette-specific “outward” and gene-specific “inward” primers
C:Identification of single-copy targeted transformants with external gene-specific primers
D: Southern blot (*Hind*III digest) to identify transformants containing only a single, targeted selection cassette.

In Supporting Figures S1 Fig – S7 Fig, Red boxes = protein-coding sequence, Blue boxes = 5’- and 3’-UTRs, Green boxes = *NPTII* selection cassette, Red triangles = *LoxP* sites. In Southern blots, the *NPTII* sequence was used as a probe.
