# Supplementary material for: The Transcriptional Response to DNA-Double-Strand Breaks in Physcomitrella patens
Source: PLoS One. 2016 Aug 18;11(8):e0161204. doi: 10.1371/journal.pone.0161204 (PMC4990234; doi:10.1371/journal.pone.0161204)

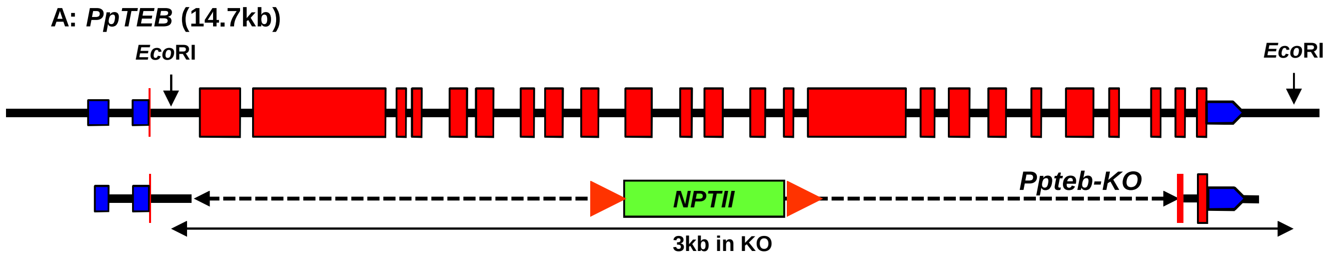

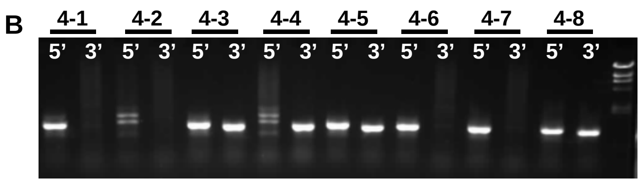

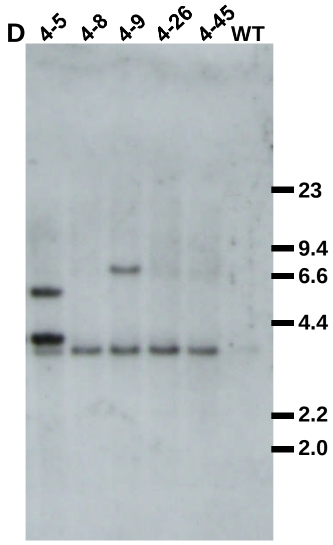

S2 Fig: *Ppteb-KO*

A: Schematic of gene structure and knockout construct.

B: Identification of targeted loci by PCR amplification with cassette-specific “outward” and gene-specific “inward” primers

C:Identification of single-copy targeted transformants with external gene-specific primers (track “P” = plasmid control).

D: Southern blot (*Eco*RI digest) to identify transformants containing only a single, targeted selection cassette

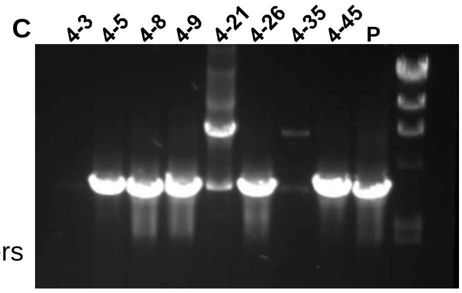

Supplement: S2 Fig — A: Schematic of gene structure and knockout construct. B: Identification of targeted loci by PCR amplification with cassette-specific “outward” and gene-specific “inward” primers. C: Identification of single-copy targeted transformants with external gene-specific primers (track “P” = plasmid control). D: Southern blot (EcoRI digest) to identify transformants containing only a single, targeted selection cassette. (PDF) [file pone.0161204.s004.pdf]
