# Supplementary material for: The Transcriptional Response to DNA-Double-Strand Breaks in Physcomitrella patens
Source: PLoS One. 2016 Aug 18;11(8):e0161204. doi: 10.1371/journal.pone.0161204 (PMC4990234; doi:10.1371/journal.pone.0161204)
Supplement: S3 Fig — A: Schematic of gene structure and knockout construct. B: Identification of targeted loci by PCR amplification with cassette-specific “outward” and gene-specific “inward” primers. C: Identification of single-copy targeted transformants with external gene-specific primers (Track “P” = plasmid control). D: Southern blot (HindIII digest) to identify transformants containing only a single, targeted selection cassette. (Analysed on same gel as PptebKO: see S2 Fig for WT control). (PPTX) [file pone.0161204.s005.pptx]

## Slide 1
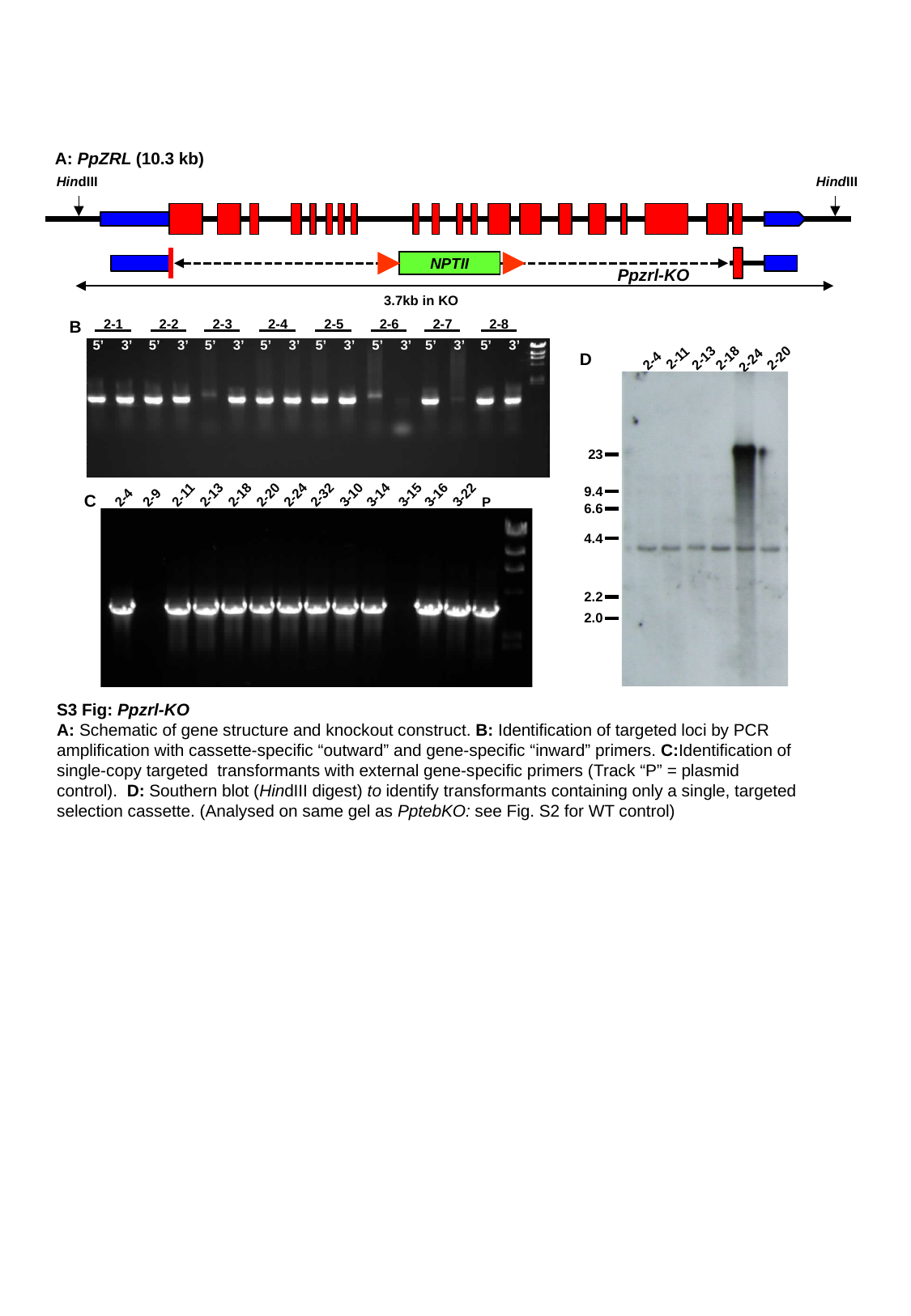

A: PpZRL (10.3 kb)
NPTII
Ppzrl-KO
HindIII
HindIII
3.7kb in KO
2-1
2-2
2-3
2-4
2-5
2-6
2-7
2-8
5’
3’
5’
3’
5’
3’
5’
3’
5’
3’
5’
3’
5’
3’
5’
3’
B
D
2-11
2-13
2-18
2-20
2-24
2-4
23
9.4
6.6
4.4
2.2
2.0
2-11
2-13
2-18
2-20
2-24
2-32
3-10
3-14
3-15
3-16
3-22
2-4
2-9
P
C
S3 Fig: Ppzrl-KO
A: Schematic of gene structure and knockout construct. B: Identification of targeted loci by PCR
amplification with cassette-specific “outward” and gene-specific “inward” primers. C:Identification of single-copy targeted transformants with external gene-specific primers (Track “P” = plasmid control). D: Southern blot (HindIII digest) to identify transformants containing only a single, targeted selection cassette. (Analysed on same gel as PptebKO: see Fig. S2 for WT control)
