# Supplementary material for: The Transcriptional Response to DNA-Double-Strand Breaks in Physcomitrella patens
Source: PLoS One. 2016 Aug 18;11(8):e0161204. doi: 10.1371/journal.pone.0161204 (PMC4990234; doi:10.1371/journal.pone.0161204)
Supplement: S4 Fig — A: Schematic of gene structure and knockout construct. B: Identification of targeted loci by PCR amplification with cassette-specific “outward” and gene-specific “inward” primers C: Identification of single-copy targeted transformants with external gene-specific primers (Tracks “P” and “wt” = plasmid and wild-type genomic DNA controls) D: Southern blot (HindIII digest) to identify transformants containing only a single, targeted selection cassette. Analysed on same gel as Ppchd5-KO: see S5 Fig for wild-type control). (PPTX) [file pone.0161204.s006.pptx]

## Slide 1
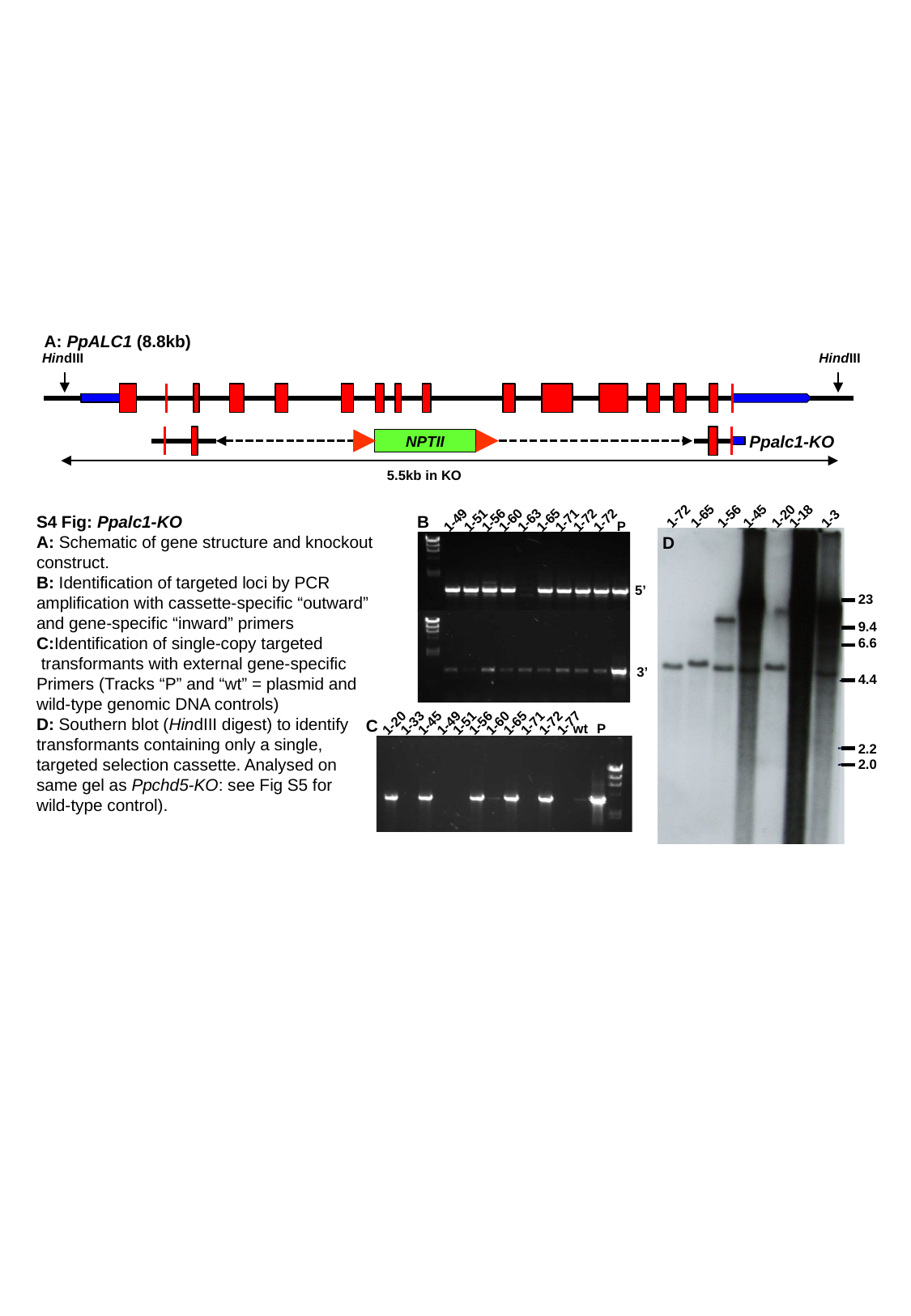

A: PpALC1 (8.8kb)
HindIII
HindIII
Ppalc1-KO
NPTII
5.5kb in KO
1-72
1-65
1-56
1-45
1-20
1-18
1-3
S4 Fig: Ppalc1-KO
A: Schematic of gene structure and knockout
construct.
B: Identification of targeted loci by PCR
amplification with cassette-specific “outward”
and gene-specific “inward” primers
C:Identification of single-copy targeted
 transformants with external gene-specific
Primers (Tracks “P” and “wt” = plasmid and
wild-type genomic DNA controls)
D: Southern blot (HindIII digest) to identify
transformants containing only a single,
targeted selection cassette. Analysed on
same gel as Ppchd5-KO: see Fig S5 for
wild-type control).
B
1-49
1-51
1-56
1-60
1-63
1-65
1-71
1-72
1-72
P
D
5’
23
9.4
6.6
3’
4.4
1-20
1-33
1-45
1-49
1-51
1-56
1-60
1-65
1-71
1-72
1-77
C
wt
P
2.2
2.0
