# Supplementary material for: The Transcriptional Response to DNA-Double-Strand Breaks in Physcomitrella patens
Source: PLoS One. 2016 Aug 18;11(8):e0161204. doi: 10.1371/journal.pone.0161204 (PMC4990234; doi:10.1371/journal.pone.0161204)
Supplement: S5 Fig — A: Schematic of gene structure and knockout construct. B: Identification of targeted loci by PCR amplification with cassette-specific “outward” and gene-specific “inward” primers C: Identification of single-copy targeted Transformants with external gene-specific primers (Tracks “P” and “wt” = plasmid and wild-type genomic DNA controls). D: Southern blot (BglII digest) to identify transformants containing only a single, targeted selection cassette. (PPTX) [file pone.0161204.s007.pptx]

## Slide 1
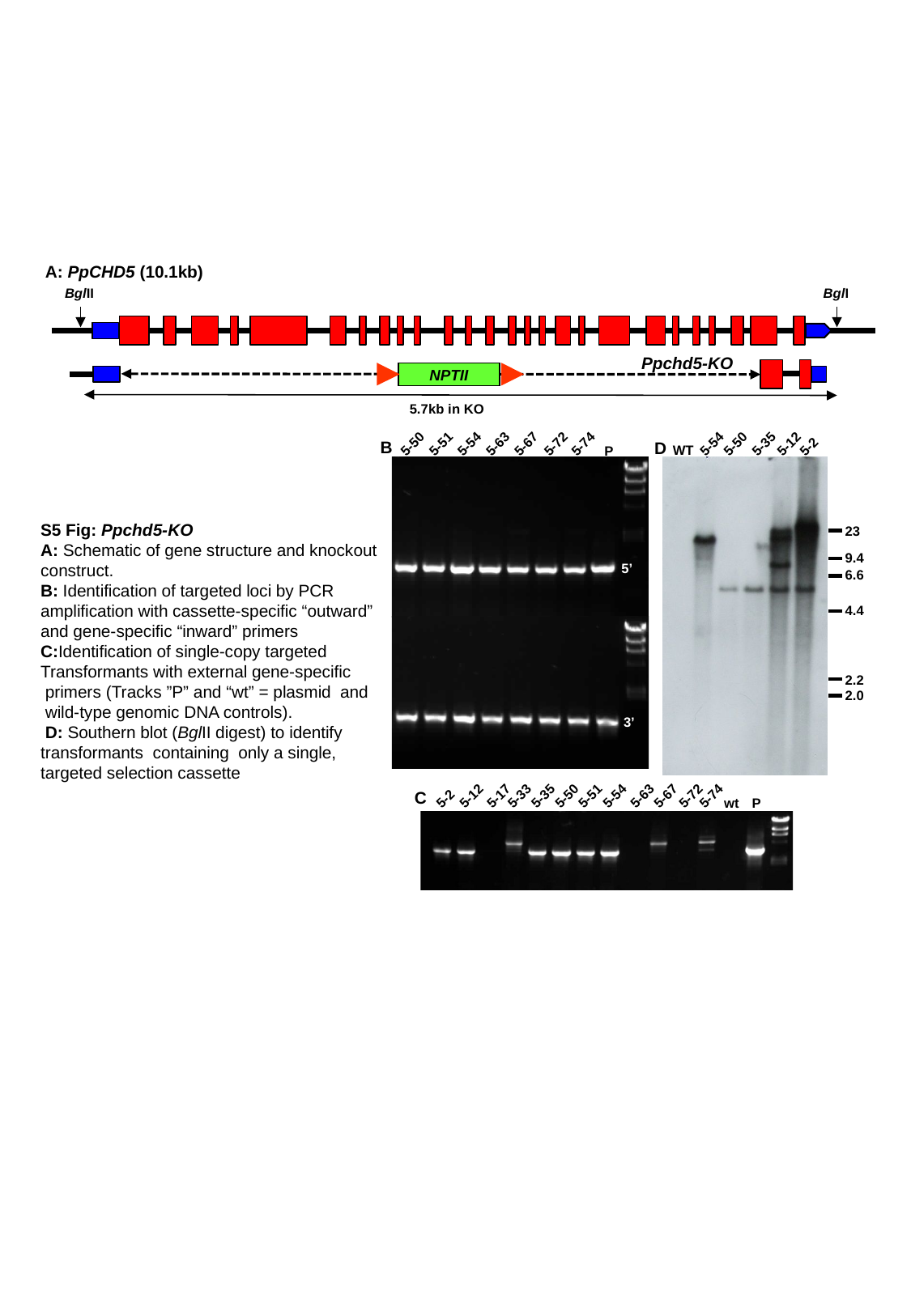

A: PpCHD5 (10.1kb)
BglII
BglI
Ppchd5-KO
NPTII
5.7kb in KO
5-12
5-54
5-50
5-35
5-2
5-50
5-51
5-54
5-63
5-67
5-72
5-74
B
D
WT
P
S5 Fig: Ppchd5-KO
A: Schematic of gene structure and knockout
construct.
B: Identification of targeted loci by PCR
amplification with cassette-specific “outward”
and gene-specific “inward” primers
C:Identification of single-copy targeted
Transformants with external gene-specific
 primers (Tracks ”P” and “wt” = plasmid and
 wild-type genomic DNA controls).
 D: Southern blot (BglII digest) to identify
transformants containing only a single,
targeted selection cassette
23
9.4
5’
6.6
4.4
2.2
2.0
3’
C
5-12
5-17
5-33
5-35
5-50
5-51
5-54
5-63
5-67
5-72
5-74
5-2
wt
P
