# Supplementary material for: The Transcriptional Response to DNA-Double-Strand Breaks in Physcomitrella patens
Source: PLoS One. 2016 Aug 18;11(8):e0161204. doi: 10.1371/journal.pone.0161204 (PMC4990234; doi:10.1371/journal.pone.0161204)
Supplement: S6 Fig — A: Schematic of gene structure and knockout construct. B: Identification of targeted loci by PCR amplification with cassette-specific “outward” and gene-specific “inward” primers. C: Identification of single-copy targeted transformants with external gene-specific primers (Track “P” = plasmid control). D: Southern blot (EcoRV digest) to identify transformants containing only a single, targeted selection cassette. (PPTX) [file pone.0161204.s008.pptx]

## Slide 1
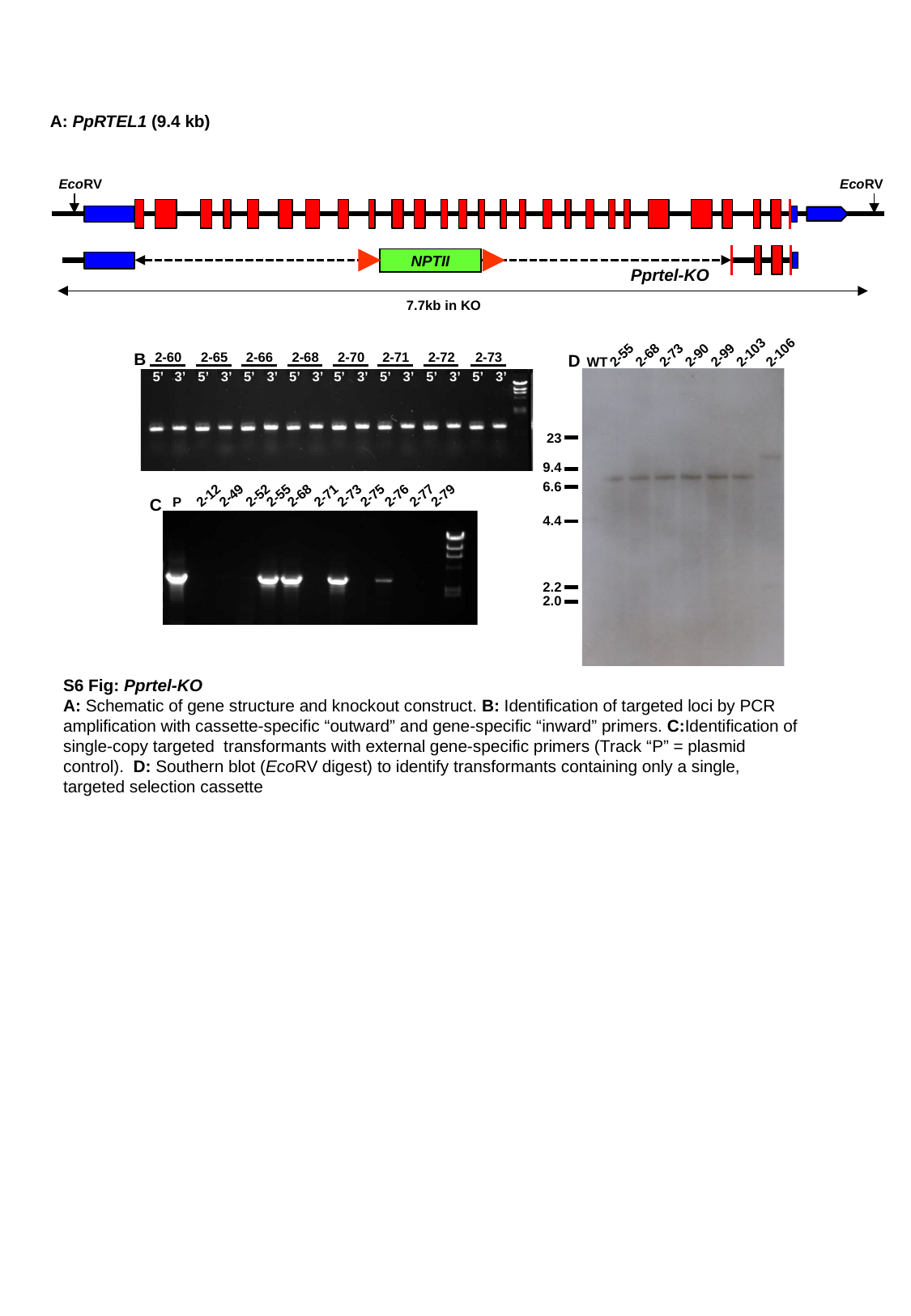

A: PpRTEL1 (9.4 kb)
EcoRV
EcoRV
NPTII
Pprtel-KO
7.7kb in KO
2-103
2-106
2-55
2-68
2-73
2-90
2-99
B
2-60
2-65
2-66
2-68
2-70
2-71
2-72
2-73
D
WT
5’
3’
5’
3’
5’
3’
5’
3’
5’
3’
5’
3’
5’
3’
5’
3’
23
9.4
6.6
2-12
2-49
2-52
2-55
2-68
2-71
2-73
2-75
2-76
2-77
2-79
P
C
4.4
2.2
2.0
S6 Fig: Pprtel-KO
A: Schematic of gene structure and knockout construct. B: Identification of targeted loci by PCR
amplification with cassette-specific “outward” and gene-specific “inward” primers. C:Identification of single-copy targeted transformants with external gene-specific primers (Track “P” = plasmid control). D: Southern blot (EcoRV digest) to identify transformants containing only a single, targeted selection cassette
