# Supplementary material for: The Transcriptional Response to DNA-Double-Strand Breaks in Physcomitrella patens
Source: PLoS One. 2016 Aug 18;11(8):e0161204. doi: 10.1371/journal.pone.0161204 (PMC4990234; doi:10.1371/journal.pone.0161204)
Supplement: S7 Fig — A: Schematic of gene structure and knockout construct. B: Identification of targeted loci by PCR amplification with cassette-specific “outward” and gene-specific “inward” primers C: Identification of single-copy targeted transformants with external gene-specific Primers primers (Tracks “P” and “wt” = plasmid and wild-type genomic DNA controls). D: Southern blot (BglII digest) to identify transformants containing only a single, targeted selection cassette (PPTX) [file pone.0161204.s009.pptx]

## Slide 1
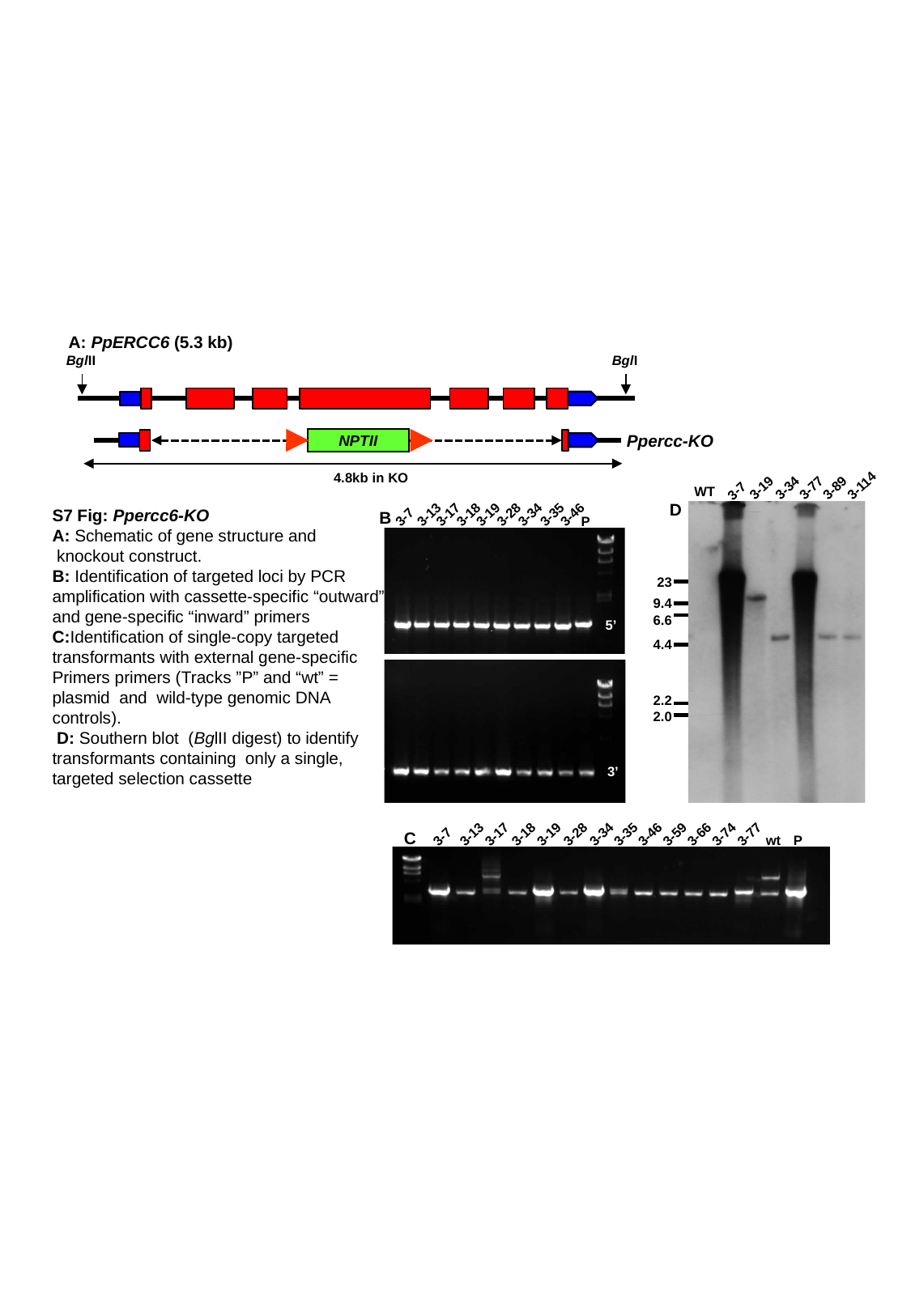

A: PpERCC6 (5.3 kb)
Ppercc-KO
NPTII
BglII
BglI
4.8kb in KO
3-114
3-19
3-34
3-77
3-89
3-7
23
9.4
6.6
4.4
2.2
2.0
D
3-46
3-13
3-17
3-18
3-19
3-28
3-34
3-35
3-7
P
S7 Fig: Ppercc6-KO
A: Schematic of gene structure and
 knockout construct.
B: Identification of targeted loci by PCR
amplification with cassette-specific “outward”
and gene-specific “inward” primers
C:Identification of single-copy targeted
transformants with external gene-specific
Primers primers (Tracks ”P” and “wt” =
plasmid and wild-type genomic DNA
controls).
 D: Southern blot (BglII digest) to identify
transformants containing only a single,
targeted selection cassette
B
5’
3’
3-46
3-13
3-17
3-18
3-19
3-28
3-34
3-35
3-59
3-66
3-74
3-77
3-7
wt
P
C
WT
