# Supplementary material for: The Transcriptional Response to DNA-Double-Strand Breaks in Physcomitrella patens
Source: PLoS One. 2016 Aug 18;11(8):e0161204. doi: 10.1371/journal.pone.0161204 (PMC4990234; doi:10.1371/journal.pone.0161204)
Supplement: S8 Fig — (PPTX) [file pone.0161204.s010.pptx]

## Slide 1
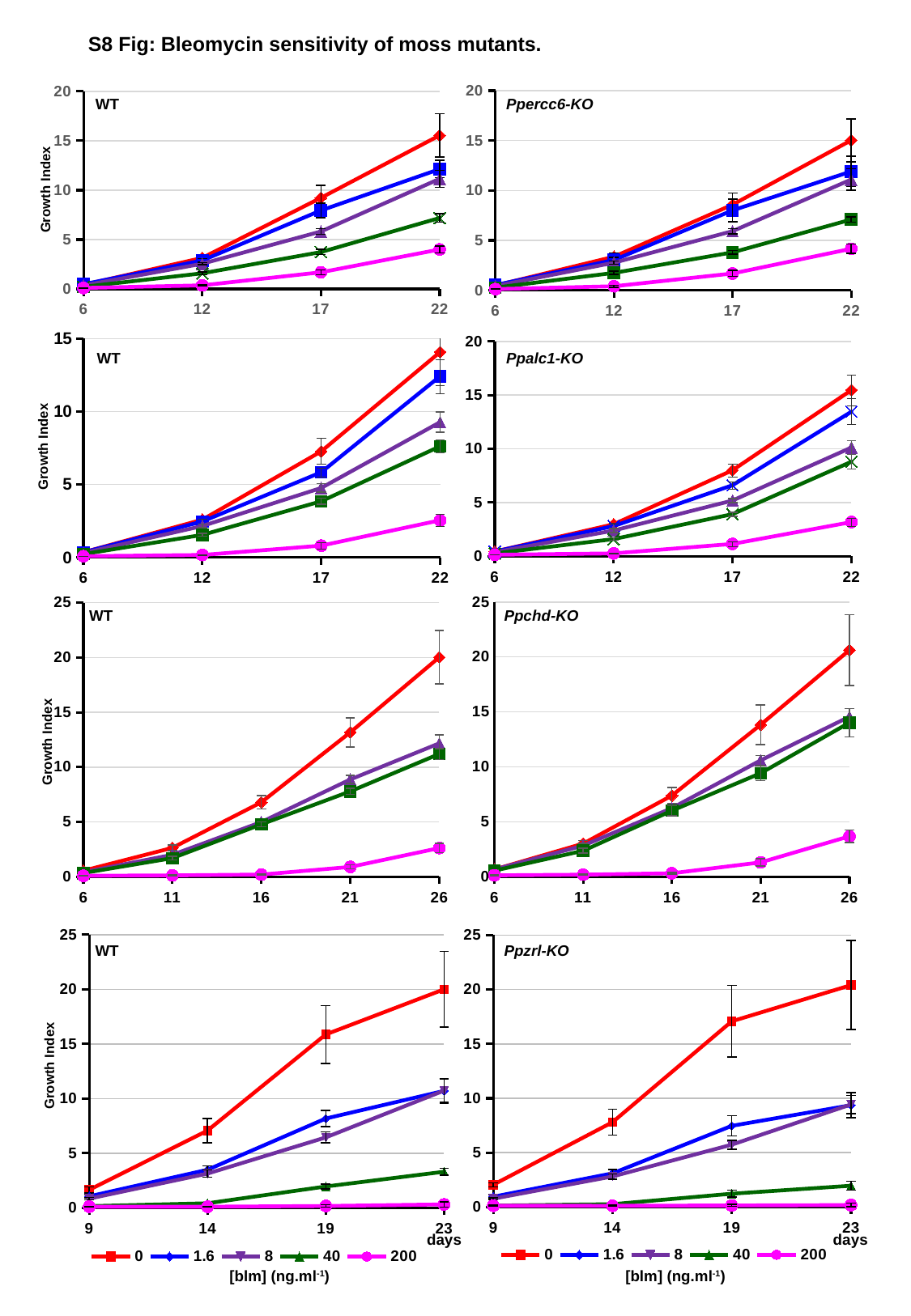

S8 Fig: Bleomycin sensitivity of moss mutants.
### Chart
| Category | | | | | |
|---|---|---|---|---|---|
| 6 | 0.47743140455392763 | 0.5240395285189798 | 0.42858947153873705 | 0.26576290374891115 | 0.12195389949404829 |
| 12 | 3.3612805274246504 | 3.0340103721480958 | 2.7642748988444774 | 1.745311245524568 | 0.4041866944006048 |
| 17 | 8.575767877310318 | 8.006524058983315 | 5.922503578586083 | 3.797610508803832 | 1.681546672877286 |
| 22 | 15.02953859575507 | 11.905820241287183 | 11.099139208337185 | 7.086595973173456 | 4.158280408040377 |
### Chart
| Category | | | | | |
|---|---|---|---|---|---|
| 6 | 0.4177222923759011 | 0.47525796872136045 | 0.36193743934000966 | 0.2173435832567197 | 0.09889133038180747 |
| 12 | 3.1392499690001356 | 2.88151014367543 | 2.55307374484537 | 1.5838698164750273 | 0.3634237054352237 |
| 17 | 9.227341088204309 | 7.943335463793588 | 5.818155356102851 | 3.732912236615708 | 1.6873810355588925 |
| 22 | 15.546464021468823 | 12.131626922320132 | 11.148982625331596 | 7.182786063282767 | 3.998971127563587 |WT
Ppercc6-KO
Growth Index
### Chart
| Category | | | | | |
|---|---|---|---|---|---|
| 6 | 0.3588978876703072 | 0.3574734832718557 | 0.3085340667024701 | 0.23338295349281468 | 0.08952535415440702 |
| 12 | 2.59020665736947 | 2.466128531964644 | 2.1598507567008047 | 1.5502471015153056 | 0.1749048923444578 |
| 17 | 7.2709027341183035 | 5.835940340903003 | 4.764167077059759 | 3.8506385141345367 | 0.8135830221073685 |
| 22 | 14.076057135777123 | 12.418061096465879 | 9.273016902321778 | 7.621297774709072 | 2.550678771304115 |
### Chart
| Category | | | | | |
|---|---|---|---|---|---|
| 6 | 0.43427713617305785 | 0.4339916466924059 | 0.3696281371534849 | 0.2536575343947001 | 0.13339098359479282 |
| 12 | 2.9721350378932865 | 2.80754404848382 | 2.3880092794450976 | 1.5909505100153836 | 0.2591121954141967 |
| 17 | 7.99667729666222 | 6.606612023692752 | 5.181331064462702 | 3.9111919290948345 | 1.1419190399490238 |
| 22 | 15.46710444802379 | 13.458332745957682 | 10.120159437247136 | 8.793099728131878 | 3.17297390642401 |WT
Ppalc1-KO
Growth Index
### Chart
| Category | | | | |
|---|---|---|---|---|
| 6 | 0.6731848814325235 | 0.6707577708871092 | 0.5630772240153135 | 0.13533247508781251 |
| 11 | 3.0219679363764915 | 2.8391651319828117 | 2.3718948703212095 | 0.19894443660830263 |
| 16 | 7.39011764480741 | 6.248737628761866 | 6.016438696960563 | 0.32572220949326003 |
| 21 | 13.834871482196581 | 10.596590822009766 | 9.42552423829041 | 1.3148129785539653 |
| 26 | 20.62173315956895 | 14.570110205886067 | 14.029798231176317 | 3.6869326444484427 |
### Chart
| Category | | | | |
|---|---|---|---|---|
| 6 | 0.5425470232535388 | 0.4070031487039463 | 0.3290438864903383 | 0.08015623685072579 |
| 11 | 2.6280734919552824 | 1.996831650409009 | 1.6882207349926772 | 0.11643103271226646 |
| 16 | 6.783294820088126 | 4.9762318609699 | 4.772293427870194 | 0.1913521021881086 |
| 21 | 13.17293371230201 | 8.868808054675997 | 7.783318090416671 | 0.8882911859823519 |
| 26 | 20.02301677386808 | 12.169050312432663 | 11.20067249990102 | 2.60996437513118 |WT
Ppchd-KO
Growth Index
### Chart
| Category | | | | | |
|---|---|---|---|---|---|
| 9 | 1.6377104908093711 | 1.0470062610148911 | 0.8381849165045822 | 0.15217324642099916 | 0.10009118196686871 |
| 14 | 7.06787608832763 | 3.479958329034212 | 3.1289581732274496 | 0.4133813059787185 | 0.09816635154442893 |
| 19 | 15.86677730705536 | 8.168302558592856 | 6.439251475846706 | 1.947972470827742 | 0.16608209304750646 |
| 23 | 20.00299338526154 | 10.707698821638225 | 10.734114039861927 | 3.300242009393974 | 0.30714911084638236 |
### Chart
| Category | | | | | |
|---|---|---|---|---|---|
| 9 | 2.045495365259598 | 0.9470660050357561 | 0.765137377560497 | 0.12786057228968442 | 0.11680970906463141 |
| 14 | 7.79986742637429 | 3.095644629648737 | 2.8111924686192467 | 0.2601107009841937 | 0.11257508213526389 |
| 19 | 17.07491821271587 | 7.459386599627022 | 5.729258543047239 | 1.2252150276313147 | 0.14437598644036642 |
| 23 | 20.39901563585385 | 9.363323429927421 | 9.433056637867752 | 1.9555862641768818 | 0.1768379908046637 |WT
Ppzrl-KO
Growth Index
days
days
[blm] (ng.ml-1)
[blm] (ng.ml-1)

## Slide 2
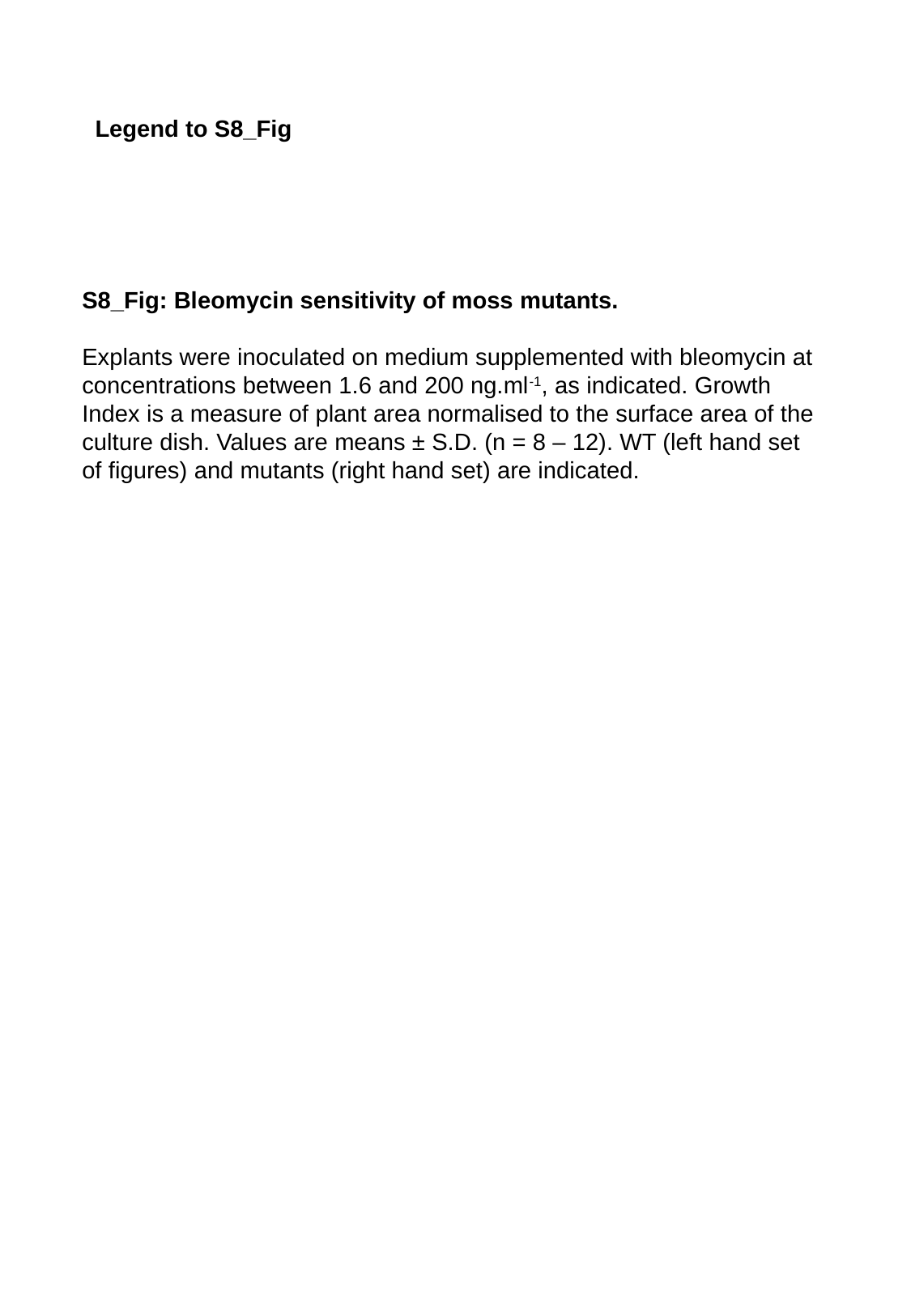

Legend to S8_Fig
S8_Fig: Bleomycin sensitivity of moss mutants.
Explants were inoculated on medium supplemented with bleomycin at concentrations between 1.6 and 200 ng.ml-1, as indicated. Growth Index is a measure of plant area normalised to the surface area of the culture dish. Values are means ± S.D. (n = 8 – 12). WT (left hand set of figures) and mutants (right hand set) are indicated.
