# Supplementary material for: The Transcriptional Response to DNA-Double-Strand Breaks in Physcomitrella patens
Source: PLoS One. 2016 Aug 18;11(8):e0161204. doi: 10.1371/journal.pone.0161204 (PMC4990234; doi:10.1371/journal.pone.0161204)
Supplement: S9 Fig — Each photograph represents the final time-point presented in the growth test analysis in Fig 4. For Ppteb-KO the growth on 40ng.ml-1 bleomycin is shown. For all other mutants, growth on 200ng.ml-1 bleomycin is shown. (PPTX) [file pone.0161204.s011.pptx]

## Slide 1
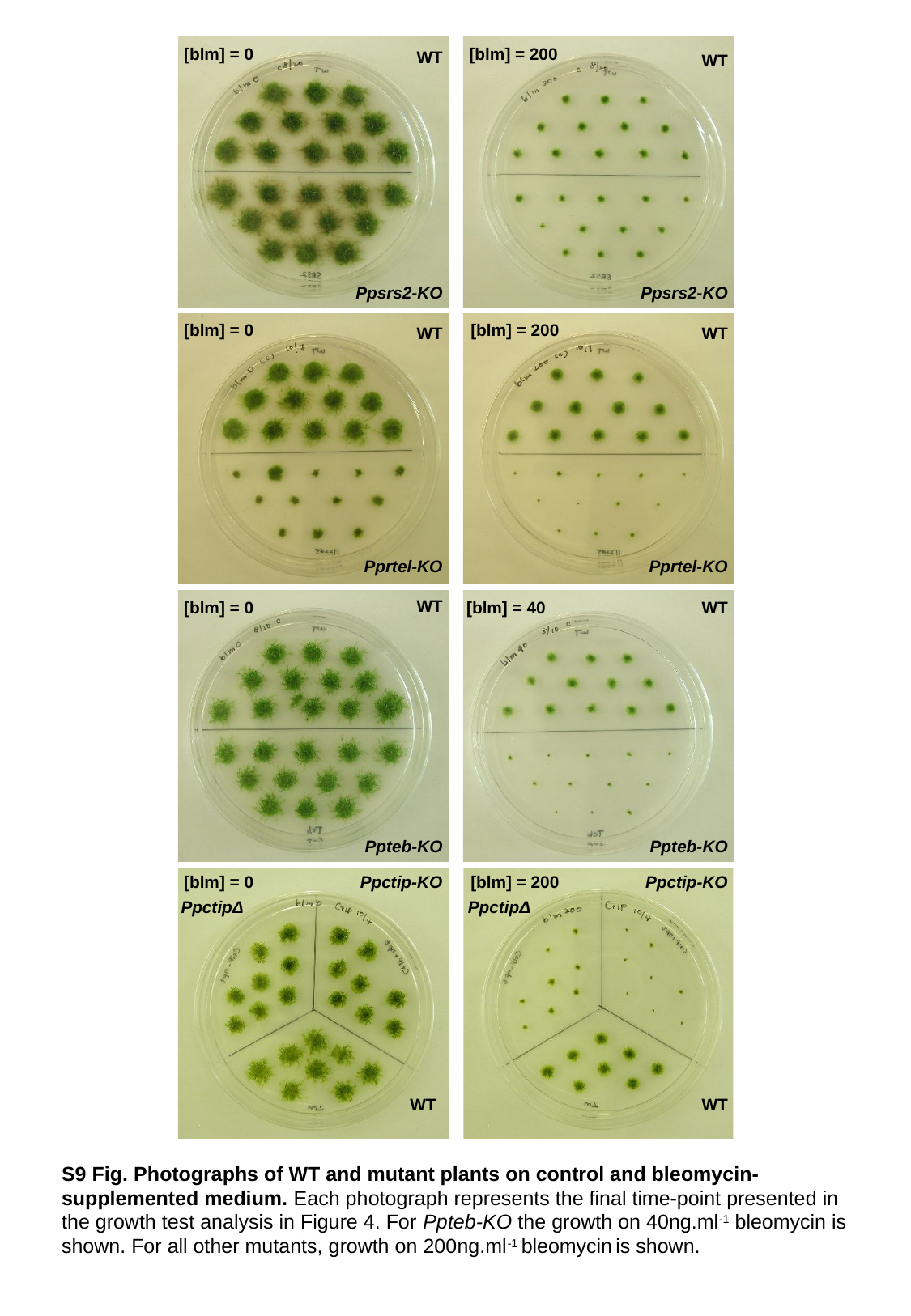

[blm] = 0
[blm] = 200
WT
WT
Ppsrs2-KO
Ppsrs2-KO
[blm] = 0
[blm] = 200
WT
WT
Pprtel-KO
Pprtel-KO
WT
[blm] = 0
[blm] = 40
WT
Ppteb-KO
Ppteb-KO
[blm] = 0
Ppctip-KO
[blm] = 200
Ppctip-KO
PpctipΔ
PpctipΔ
WT
WT
S9 Fig. Photographs of WT and mutant plants on control and bleomycin-supplemented medium. Each photograph represents the final time-point presented in the growth test analysis in Figure 4. For Ppteb-KO the growth on 40ng.ml-1 bleomycin is shown. For all other mutants, growth on 200ng.ml-1 bleomycin is shown.
