# Supplementary material for: The Transcriptional Response to DNA-Double-Strand Breaks in Physcomitrella patens
Source: PLoS One. 2016 Aug 18;11(8):e0161204. doi: 10.1371/journal.pone.0161204 (PMC4990234; doi:10.1371/journal.pone.0161204)
Supplement: S1 Table — (PDF) [file pone.0161204.s012.pdf]

S1 Table: Primers used to construct gene targeting vectors

Primer	Sequence	Notes
PpTeb_A4	CGTGCAGCTTAAGACACAACTGA	Amplification of 5'-targeting fragment
PpTeb_S4	GATAAACGGGTCCTGAAGCATGT	Amplification of 3'-targeting fragment
PpTeb_A5	GGCAACATACTGTCCTCTTGCAC	Amplification of 3'- targeting fragment; external GSP
cPpTeb_S1	TGGAAATTGTATGGTGCGTTCA	Amplification of 5'- targeting fragment; external GSP
PpTeb_KOS	GCTCTGCTTTCAGACACCGTAAG	Amplification of knockout vector (PpTEB)
PpTeb_KOA	ACTCATGTTGTGGCAACCTTGAT	Amplification of knockout vector (PpTEB)
80443_S4	AGCTGATCCATGAGGACAGTCAC	Amplification of 5'- targeting fragment; external GSP
80443_A4	TCCTCGCTCATCCCCATATCTTA	Amplification of 5'-targeting fragment
80443_S5	CAGTAAATCCCACCACTGAGCAC	Amplification of 3'-targeting fragment
80443_A5	TGGATGGCAAAACACTTACTTGG	Amplification of 3'- targeting fragment; external GSP
80443_KOS	TTTGAAATGCCGAATAGCTTGTG	Amplification of knockout vector (PpZRL)
80443_KOA	TCCCCTTAATAGTGGCTCCATGT	Amplification of knockout vector (PpZRL)
112245_S3	AACCGAGACACCACAAATCTCCT	Amplification of 5'- targeting fragment; external GSP
112245_A3	AAGCATTCCGTCCCTAACAAAAC	Amplification of 5'-targeting fragment
112245_S4	TGGAGTTCCACTAGGTTTTGTCG	Amplification of 3'-targeting fragment
QHel112245A	TCGCTTCAAGAAAATGATGTGT	Amplification of 3'- targeting fragment; external GSP
c112245_S1	AATCGGGGAAGAATTTATGGTCA	Amplification of knockout vector (PpRTEL1)
c112245_A2	CCAGCTCCATGAAAAGTGGTATG	Amplification of knockout vector (PpRTEL1)
168107_S4	CAAACCTCCACCACTTTTAAGCA	Amplification of 5'- targeting fragment; external GSP
167108_A4	ACGTCTTCACTCGACAATTCAGC	Amplification of 5'-targeting fragment
SQHel168107F	TGGGTCACATCTTCGATAATCA	Amplification of 3'-targeting fragment
167108_A5	TTCCATTGTTCACCCTCATCAAC	Amplification of 3'- targeting fragment; external GSP
168107_KOS	CATGTCAGAAGAAAACCCCAAG	Amplification of knockout

		vector (PpCHD5)
QHel168107A	TGTAGGTAATAGCACGCTCCAT	Amplification of knockout vector (PpCHD5)
10437_S1	CACTATTGATAACCGAGCCAACG	Amplification of 5'- targeting fragment; external GSP
10437_A1	CCAAATTCATCGCAGCTCAACTA	Amplification of 5'-targeting fragment
10437_S2	GAAAGGATCGCTGGAGTCAAAGT	Amplification of 3'-targeting fragment
10437_A2	AGCACCAAAACTCCTGCTTGATT	Amplification of 3'- targeting fragment; external GSP
10437_KOS	CTGGTCAATCAAACCTCCACATC	Amplification of knockout vector (PpERCC6)
10437_KOA	GCAGTGAGGTTGGCAAAACATTA	Amplification of knockout vector (PpERCC6)
5’108610-F	GGGTATTACTGCGGAGCTGAAG	Amplification of 5'- targeting fragment; external GSP
5’108610-R	AGCCGCTTTGTATCGAGTGAAG	Amplification of 5'-targeting fragment
PpAlc1_S2	CAGAATGATGGGTCACTGAAACC	Amplification of 3'-targeting fragment
QHel108610A	TTCATCATTTCAACTCCTGTCG	Amplification of 3'- targeting fragment; external GSP
Alc1_KOS	TGTGGGTCGTACTGTGGAAGTTT	Amplification of knockout vector (PpALC1)
Alc1_KOA	CCCAAATGTGACAGAAACAGCA	Amplification of knockout vector (PpALC1)
CtIP-S	AACACAGTCTTCCATCCTCTGCAC	Amplification of CtIP gene; 5'-external GSP
CtIP-A	CTCCATACGCAAAAGGAAGCAAT	Amplification of CtIP gene; 3'-external GSP
CtIP-KOS	AAGGCACATAGCTCCACAAGCAT	Amplification of knockout vector (PpCtIP)
CtIP-KOA	GACAAGCCGTCACCGTAACTACA	Amplification of knockout vector (PpCtIP1)
