# Supplementary material for: The Transcriptional Response to DNA-Double-Strand Breaks in Physcomitrella patens
Source: PLoS One. 2016 Aug 18;11(8):e0161204. doi: 10.1371/journal.pone.0161204 (PMC4990234; doi:10.1371/journal.pone.0161204)
Supplement: S2 Table — (PDF) [file pone.0161204.s013.pdf]

S2 Table: Primers used for real-time quantitative PCR.

Gene	Primer sequence	Notes
PpRad51A	CTGTTATTTGGCATCACCTTCA	Forward
	TTGACCAAGAACAGACAACTGG	Reverse
PpRad51B	GCCTGTGACATTGTATTGGAGA	Forward
	GGTTGTACCATTACCCTCCTGA	Reverse
PpKu70	ATCTTGGCAAATGAGGTCAAAG	Forward
	GCTCTCTGCTCAAAACTCCAAT	Reverse
PpKu80	GCTAACTTTCATGGCCAACTCT	Forward
	TCTGGCCTTTGAAGTCTCTGAT	Reverse
PpPARP-2	AGAAGTGTTGCGTCCTGTTTTT	Forward
	ATGTCGGGGGTTCTATTTCAGT	Reverse
PpPARP-3	GTTTCGGGTAGAAATGGTCAAA	Forward
	CCAGGATGCACTTATATGCTTG	Reverse
PpSrs2	CATAGATCATCATTTGGGTTCG	Forward
	GAGCCTTGTTTGCTTTGGTTAC	Reverse
PpAlc1	AGACAAATTCAAGCGTCTTGGT	Forward
	TTCATCATTTCAACTCCTGTCG	Reverse
PpCAP-50	GCAACAGTAGTCGGTGTCTTCA	Forward
	TATCCGAAATAGGACACCTTGC	Reverse
