# Supplementary material for: The Transcriptional Response to DNA-Double-Strand Breaks in Physcomitrella patens
Source: PLoS One. 2016 Aug 18;11(8):e0161204. doi: 10.1371/journal.pone.0161204 (PMC4990234; doi:10.1371/journal.pone.0161204)
Supplement: S3 Table — (PDF) [file pone.0161204.s014.pdf]

S3 Table: Down-regulated genes that are up-regulated by drought-stress

Fold change	V3.3 ID	Phypa1_1:ID and annotation
3x	Pp3c13_6860V3.1	228680: Group 2 LEA protein
4x	Pp3c20_8030V3.1	228642: Unknown drought-induced protein
4x	Pp3c12_22320V3.1	166566: Group 3 LEA protein
4x	Pp3c10_6760V3.1	228663: TspO-like protein
4x	Pp3c11_4920V3.1	228732: Unknown drought-induced protein
4x	Pp3c21_4870V3.1	166706: Unknown drought-induced protein
