# Supplementary material for: The Transcriptional Response to DNA-Double-Strand Breaks in Physcomitrella patens
Source: PLoS One. 2016 Aug 18;11(8):e0161204. doi: 10.1371/journal.pone.0161204 (PMC4990234; doi:10.1371/journal.pone.0161204)
Supplement: S4 Table — (PDF) [file pone.0161204.s015.pdf]

S4 Table: Up-regulated genes annotated as transcription factors.

Fold change	V3.3 ID	Phypa1_1:ID and annotation
n.c.	Pp3c2_31770V3.1	61394: R2R3 Myb family transcription factor
310x	Pp3c23_5390V3.1	97669: Myb-like transcription factor
49x	Pp3c7_20200V3.1	173967: AP2/EREBP transcription factor
42x	Pp3c11_14690V3.1	159296: AP2/EREBP transcription factor
34x	Pp3c6_26080V3.1	163201: AP2/EREBP transcription factor
27x	Pp1s109_122V2.1	233888: WRKY transcription factor
17x	Pp3c19_3000V3.1	234503: GATA transcription factor
14x	Pp3c24_7690V3.1	233209: Myb-like transcription factor
13x	Pp3c2_32530V3.1	49725: NAC/NAM transcription factor
12x	Pp3c7_10780V3.1	165365: AP2/EREBP transcription factor
12x	Pp3c7_20170V3.1	158560: AP2/EREBP transcription factor
11x	Pp3c5_2900V3.1	128717: AP2/EREB transcription factor
9x	Pp3c14_21620V3.1	206659: HD- Zip transcription factor
8x	Pp3c13_2080V3.1	166279: bHLH family transcription factor
8x	Pp3c12_15190V3.1	164510: NAC/NAM transcription factor
7x	Pp3c17_23620V1.1	78012: Myb-like transcription factor
7x	Pp3c13_6470V3.1	168588: NAC/NAM transcription factor
7x	Pp3c2_25760V3.1	166576: AP2/EREBP transcription factor
6x	Pp3c23_9520V3.1	114556: NAC/NAM transcription factor
6x	Pp3c11_10350V3.1	235529: R2R3 Myb transcription factor
6x	Pp3c20_18130V3.1	208168: NAC/NAM transcription factor
6x	Pp3c13_24020V3.1	72578: Ap2/EREBP transcription factor
5x	Pp3c27_5180V3.1	Unannotated Ap2/EREBP transcription factor
5x	Pp3c16_13260V3.1	176040: AP2/EREBP transcription factor
5x	Pp3c4_26880V3.1	19197: WRKY transcription factor
5x	Pp3c1_7800V3.1	207319: Ap2/EREBP transcription factor
4x	Pp3c2_26970V3.1	160508: bZIP transcription factor
4x	Pp3c1_41530V3.1	233952: Myb-like transcription factor
4x	Pp3c6_2730V3.1	184805: HD-Zip transcription factor
4x	Pp3c1_14230V3.1	233473: AP2/EREBP transcription factor
4x	Pp3c25_4250V3.1	198785: AP2/EREBP transcription factor
4x	Pp3c14_17020V3.1	124219: WRKY transcription factor
4x	Pp3c19_6370V3.1	149195: AP2/EREBP transcription factor
3x	Pp3c17_20210V3.1	77514: B3 family transcription factor
3x	Pp3c18_11980V3.1	64872: AP2/EREBP transcription factor
3x	Pp3c17_10170V3.1	19961: AP2/EREBP transcription factor
3x	Pp3c22_1790V3.1	87858: AP2/EREBP transcription factor
