# Supplementary material for: The Transcriptional Response to DNA-Double-Strand Breaks in Physcomitrella patens
Source: PLoS One. 2016 Aug 18;11(8):e0161204. doi: 10.1371/journal.pone.0161204 (PMC4990234; doi:10.1371/journal.pone.0161204)
Supplement: S5 Table — (PDF) [file pone.0161204.s016.pdf]

S5 Table: Down-regulated genes annotated as transcription factors

Fold change	V3.3 ID	Phypa1_1:ID and annotation
3x	Pp3c9_5650V3.1	226592: Histone-like transcription factor, CCAAT-binding factor, subunit C
3x	p3c23_6860V3.1	66431:NAC/NAM transcription factor
3x	Pp3c4_11507V3.1	92601: NAC/ NAM transcription factor
3x	Pp3c4_11490V3.1	145456: NAC/NAM transcription factor
3x	Pp3c8_16910V3.1	166399: Myb family transcription factor
4x	Pp3c23_15540V3.1	110184: Transcription factor containing NAC, ubiquitin-associated and TS-N domains
4x	Pp3c1_36120V3.1	70478: Ap2/EREBP transcription factor
4x	Pp3c16_19170V3.1	235399: MADS-domain transcription factor
4x	Pp3c21_11230V3.1	107983: NAC/NAM transcription factor
4x	Pp1s121_54V2.1	84228: bHLH transcription factor
5x	Pp3c22_10770V3.1	183238: AP2/EREBP transcription factor
6x	Pp3c5_21370V3.1	92067: Myb family transcription factor
7x	Pp3c27_1350V3.1	228103:AP2/EREBP transcription factor
12x	Pp3c16_3770V3.1	189022: Ap2/EREBP transcription factor.
